# Investigating the Growth of Algae Under Low Atmospheric Pressures for Potential Food and Oxygen Production on Mars

**DOI:** 10.3389/fmicb.2021.733244

**Published:** 2021-11-12

**Authors:** Leena M. Cycil, Elisabeth M. Hausrath, Douglas W. Ming, Christopher T. Adcock, James Raymond, Daniel Remias, Warren P. Ruemmele

**Affiliations:** ^1^Department of Geoscience, University of Nevada, Las Vegas, Las Vegas, NV, United States; ^2^NASA Johnson Space Center, Houston, TX, United States; ^3^School of Life Sciences, University of Nevada, Las Vegas, Las Vegas, NV, United States; ^4^School of Engineering, University of Applied Sciences Upper Austria, Wels, Austria

**Keywords:** BLSS, life on mars, space biology, low pressure chamber, extremophilic algae

## Abstract

With long-term missions to Mars and beyond that would not allow resupply, a self-sustaining Bioregenerative Life Support System (BLSS) is essential. Algae are promising candidates for BLSS due to their completely edible biomass, fast growth rates and ease of handling. Extremophilic algae such as snow algae and halophilic algae may also be especially suited for a BLSS because of their ability to grow under extreme conditions. However, as indicated from over 50 prior space studies examining algal growth, little is known about the growth of algae at close to Mars-relevant pressures. Here, we explored the potential for five algae species to produce oxygen and food under low-pressure conditions relevant to Mars. These included *Chloromonas brevispina*, *Kremastochrysopsis austriaca*, *Dunaliella salina*, *Chlorella vulgaris*, and *Spirulina plantensis*. The cultures were grown in duplicate in a low-pressure growth chamber at 670 ± 20 mbar, 330 ± 20 mbar, 160 ± 20 mbar, and 80 ± 2.5 mbar pressures under continuous light exposure (62–70 μmol m^–2^ s^–1^). The atmosphere was evacuated and purged with CO_2_ after sampling each week. Growth experiments showed that *D. salina, C. brevispina*, and *C. vulgaris* were the best candidates to be used for BLSS at low pressure. The highest carrying capacities for each species under low pressure conditions were achieved by *D. salina* at 160 mbar (30.0 ± 4.6 × 10^5^ cells/ml), followed by *C. brevispina* at 330 mbar (19.8 ± 0.9 × 10^5^ cells/ml) and *C. vulgaris* at 160 mbar (13.0 ± 1.5 × 10^5^ cells/ml). *C. brevispina, D. salina*, and *C. vulgaris* all also displayed substantial growth at the lowest tested pressure of 80 mbar reaching concentrations of 43.4 ± 2.5 × 10^4^, 15.8 ± 1.3 × 10^4^, and 57.1 ± 4.5 × 10^4^ cells per ml, respectively. These results indicate that these species are promising candidates for the development of a Mars-based BLSS using low pressure (∼200–300 mbar) greenhouses and inflatable structures that have already been conceptualized and designed.

## Introduction

Human exploration of Mars is one of the key scientific and technological undertakings of our time, providing critical information enabling the discovery and settlement of another world while also facilitating the development of technologies on Earth. Future human space exploration may include returning to the moon, as well as missions to Mars (Henn, 2013; Martinez et al., 2013; National Academies of Sciences, Engineering, and Medicine, 2016), with NASA aiming to send humans to Mars by the 2030s (Miranda, 2020). Current research and planning to send crewed missions to Mars for long term space exploration has underscored the critical need for advanced Bio-regenerative Life Support Systems (BLSS), which are complex mixtures of biological and engineering systems that include atmosphere revitalization, water recycling, food production, and organic waste recycling (Revellame et al., 2021). Algae, which produce much of the oxygen on Earth, can similarly be used to recycle CO_2_ and provide O_2_ and food to astronauts (Häder, 2020), and therefore, have previously been proposed for space life support systems (Averner et al., 1984).

Since the beginning of human spaceflight missions, algae have been considered promising candidates for space life support systems due to their rapid growth rates, the fact that they are straightforward to grow, and edible biomass (Powell et al., 1961; Rangel-Yagui et al., 2004; Soletto et al., 2005; Ganzer and Messerschmid, 2009; Wells et al., 2017). In the late 1960s, a bio-regenerative system utilizing the algae *Chlorella* was first studied in ground experiments for CO_2_ cleansing and O_2_ provision (Eckart, 1996). Since then, various experiments have been conducted to study the effects of radiation, microgravity, space vacuum, and temperature extremes on algae growth for space exploration missions (Horneck et al., 2003; Thirsk et al., 2009), and it has been shown that algae are capable of surviving exposure to spaceflight conditions (Niederwieser et al., 2018).

The atmospheric pressure at the surface of Mars ranges from 1 mbar to 14 mbar depending on the location and season, which is very low compared to the 1013 mbar on average at sea level on Earth (Forget, 2009). One of the goals in space exploratory missions and on Mars is to minimize the amount of mass and energy required to launch and maintain life support systems. Low-pressure is sought by the human spaceflight programs to decrease the engineering cost associated with space vehicles, as it allows a reduction in their size and in the quantity of accompanying consumable materials (Paul and Ferl, 2006). The ability to grow photosynthetic organisms under low pressure conditions is therefore an important step toward establishing advanced life support systems for long-term space missions.

Early studies of algae growth at low pressures ranging from 250 to 500 mbar suggested that low atmospheric pressures have no inhibitory effect and might slightly stimulate growth (Orcutt et al., 1970). Some algae have also developed anoxic metabolisms to adapt to low oxygen conditions (Yang et al., 2016). Limited studies have examined cyanobacterial growth under low atmospheric pressures relevant to potential growth on Mars (Kanervo et al., 2005; Murukesan et al., 2015; Verseux et al., 2021). These studies reported the successful growth of cyanobacteria at pressures as low as 100 mbar achieved with the continuous replenishment of CO_2_ and nitrogen. However, the experiments were conducted for relatively short duration (7–10 days) and detailed observations of growth dynamics such as carrying capacities and growth trends at reduced pressures were not reported (Murukesan et al., 2015; Verseux et al., 2021). To the best of our knowledge from the existing literature, including a review of over 50 space studies examining algal growth (Niederwieser et al., 2018), few studies have examined the growth of extremophilic algae, with high nutritional potential, at low pressures relevant to Mars.

Algae are found in almost all ecosystems on Earth (Rajkumar and Yaakob, 2013; Malavasi et al., 2020). Algae are diverse organisms with specialized adaptations that enable them to survive under extreme environmental conditions including hot or cold deserts (Lewis and Lewis, 2005; Schmidt et al., 2011), hypersaline habitats (Vinogradova and Darienko, 2008; Oren, 2014a; Leena et al., 2018; Cycil et al., 2020), extreme concentrations of heavy metals (Garbayo et al., 2012; Malavasi et al., 2020), deep-sea hydrothermal vents (Edgcomb et al., 2002; Malavasi et al., 2020), and extreme elevations such as the highest volcanoes on Earth (Schmidt et al., 2018; Vimercati et al., 2019). Some preliminary studies also indicated the ability of cyanobacteria and algae to grow using Martian Regolith Simulant (MRS) demonstrating their ability for *in-situ* resource utilization (Arai et al., 2008; Cycil et al., 2021). In this study, we selected algae species that are ecologically diverse and may have adaptations to thrive under extreme environmental conditions that may help them to grow under conditions relevant to Mars. Snow algae, for example, are the primary oxygen producers in challenging high UV, low temperature, and low nutrient snow environments in lower atmospheric pressures up to 6,000 m above sea level (Painter et al., 2001; Schmidt et al., 2018; Solon et al., 2018; Vimercati et al., 2019; Hoham and Remias, 2020), and can reach concentrations of over one million cells/ml (Hoham, 2001). Halophilic algae are salt-loving algae that are the main or only primary producer in most light-exposed hypersaline environments approaching salt saturation (Banciu et al., 2020). The halophilic algae *Dunaliella salina*, similar to snow algae, are the primary oxygen producers in hypersaline environments such as The Great Salt Lake, Dead Sea, Lake Tyrell, solar salterns, and brine inclusions (Oren, 2014b).

In this study, we utilized five algae strains to study their growth under Mars-relevant low-pressure conditions: three extremophilic algae, the snow algae *Chloromonas brevispina* (Hoham et al., 1979) and *Kremastochrysopsis austriaca* (Remias et al., 2020), and the halophilic algae *Dunaliella salina* (Teodoresco, 1905), in addition to two well-studied edible algal strains *Chlorella vulgaris* and *Spirulina plantensis* that have been used in multiple spaceflights and ground-based studies (Lee et al., 2001; De Morais and Costa, 2007; Daliry et al., 2017; Niederwieser et al., 2018; Detrell et al., 2019; Häder, 2020). Edible microalgae are a source of potentially healthy and sustainable nutrients. *D. salina, C. vulgaris*, and *S. plantensis* have been reported to have commercial applications as food supplements due to their rich protein content, presence of vitamins A and B12 and the abundance of β-carotene which is an antioxidant (Mokady et al., 1989; Panahi et al., 2015; Kumudha and Sarada, 2016; Lupatini et al., 2017; Canelli et al., 2020; Sui and Vlaeminck, 2020). Some reports also indicate potential applications of snow algae metabolites in the pharmaceutical industry (Sathasivam et al., 2019; Hans et al., 2021). Therefore, these algae species also have the potential to serve as healthy food sources.

## Materials and Methods

### Algae Strains and Culturing

Xenic cultures of the snow algae *C. brevispina* (from the University of Texas Culture Collection of Algae UTEX B SNO96) were provided by James Raymond, and the snow algae *K. austriaca* was isolated and provided by Daniel Remias. The *C. brevispina* culture was first isolated from Lac Laflamme by Hoham et al. (1979), and the *K. austriaca* culture was first isolated from Tyrol, Austria by Remias et al. (2020). In these experiments, *C. brevispina* and *K. austriaca* cultures were maintained on the M1 growth medium described by Hoham et al. (1979). To prepare M1 medium, 1% v/v of trace metal solution was autoclaved and added to the M1 medium prior to adding 0.1% v/v of vitamin solution (1 mg/ml vitamin B12, 5 mg/ml biotin and 1 mg/ml thiamine-HCl), which was filter-sterilized separately using a 0.2 μm filter and then added to the autoclaved M1 medium (Harrold et al., 2018; Phillips-Lander et al., 2020).

Xenic cultures of the algae *C. vulgaris* (UTEX 2714), *S. plantensis* (UTEX LB 1926), and *D. salina* (UTEX LB 200) were purchased from the UTEX Culture Collection of Algae, University of Texas, Austin along with their recommended growth media (Table 1). *S. plantensis*, originally isolated by Lewin (1969) from Del Mar Slough, San Diego Co., California, United States was maintained on sterile Enriched Seawater Medium from UTEX (Lewin, 1979). *D. salina* (Teodoresco, 1905), originally isolated from a salt lake in Russia, was maintained on sterile 2X Erdschreiber’s Medium (2X ERD UTEX) described by Foyn (1934). Xenic cultures of *C. vulgaris* (originally isolated by González et al., 1997, from a wastewater-treatment stabilization pond, Santa Fe de Bogota, Colombia) were cultivated using the sterile Proteose Medium (UTEX), where proteose is added to a Bristol medium (Bold, 1949).

**TABLE 1:** Selected algae species and their growth conditions.

	*Chloromonas brevispina*	*Kremastochrysopsis austriaca*	*Dunaliella salina*	*Spirulina plantensis*	*Chlorella vulgaris*
Classification	Snow (Psychrophilic)	Snow (Psychrophilic)	Halophilic	Mesophilic	Mesophilic
Media	M1^b^	M1^b^	2X Erd Medium^c^	Enriched Seawater Medium^d^	Proteose Medium^*e*^
Pressures (mbar)^f^	(OD_750_, cell cts)^g^ 670 ± 20, 330 ± 20, 160 ± 20, 80 ± 2.5	(OD_750_)^g^ 670 ± 20, 330 ± 20, 160 ± 20, 80 ± 2.5	(OD_750_, cell cts)^*g*^ 670 ± 20, 330 ± 20, 160 ± 20, 80 ± 2.5	(OD_750_)^g^ 670 ± 20, 330 ± 20	(OD_750_, cell cts)^g^ 670 ± 20, 330 ± 20, 160 ± 20, 80 ± 2.5
Temperatures (°C)	4.0 ± 0.1	4.0 ± 0.1	20.8 ± 2.6 (10.0 ± 0.1 at 80 ± 2.5 mbar)	20.8 ± 2.6	20.8 ± 2.6 (10.0 ± 0.1 at 80 ± 2.5 mbar)
Light levels (^a^μmol m^–2^s^–1^)	62–70^a^	62–70^a^	62–70^a^	62–70^a^	62–70^a^
Incubation time	33–54 days	33–54 days	33–62 days	62 days	33–62 days

*^a^As measured using a handheld digital lux meter (URCERI) ± 3% accuracy, where the 62–70 μmol of photons m^–2^ s^–1^ range was chosen based on previous work by Harrold et al. (2018).*

*^b^M1 medium (Hoham et al., 1979).*

*^c^2X Erdschreiber’s Medium (modified Erdschreiber’s medium, Foyn, 1934).*

*^d^Enriched Seawater Medium (Lewin, 1979).*

*^e^Proteose Medium (Bold, 1949).*

*^f^Values of uncertainties are based on the accuracy of the gauges monitoring the pressure.*

*^g^Both cell counts (cell cts) and OD_750_ readings were measured for C. brevispina, D. salina and C. vulgaris and for K. austriaca and S. plantensis only OD_750_ readings were measured due to their minimal growth.*

### Mars-Relevant Low-Pressure Chamber Design

**FIGURE 1:**
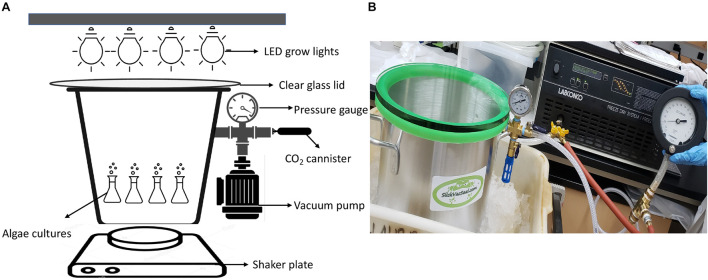
**(A)** Design of low-pressure growth chamber used for pressures of 330 mbar and below, **(B)** Photograph of the experimental setup for low-pressure growth experiments of 330 mbar and below using the SlickVacSeal aluminum vacuum chamber.

Except for the algae experiments at 670 mbar pressures, which were performed in a modified vacuum chamber (Supplementary Figure 26), all algae growth experiments were carried out in a 11.4-L (25.4 cm diameter by 23 cm tall) aluminum vacuum chamber (SlickVacSeal) (Figure 1). It is equipped with a –30–0 inch Hg (0–1014 mbar) gauge with ± 2% accuracy for routine pressure measurements. The clear tempered glass lid on the top of the chamber allowed exposure to light (Figure 1). The chamber was designed for a near full vacuum (–29.9 in Hg, ∼1 mbar) as per the manufacturer’s description. The low-pressure environment inside the chamber was generated by a Labconco direct-drive rotary vane vacuum pump (Model 117, LABCONCO). The pump has 117 (LPM) free air capacity with a vacuum to single mbar levels (SlickVacSeal, 2020). For the lowest pressure experiments at 80 mbar, instead of using the SlickVacSeal gauge, the pressure was monitored using a high sensitivity vacuum gauge (Ashcroft) that can measure –30–0 Hg (0–1014 mbar) vacuum with ± 0.25% accuracy to ensure accurate measurement of the low-pressure environment (SlickVacSeal, 2020).

The outlet on the chamber was used to establish low pressure using the vacuum pump as described above and to administer CO_2_ through a valve manifold (Figure 1). To provide a Mars-relevant atmosphere, the atmosphere within the low-pressure chamber was evacuated to the desired low-pressure and then replaced with CO_2_ using 16-gram food-grade CO_2_ threaded cartridges (ASURA). After filling the chamber with CO_2_, the chamber was evacuated again to achieve the desired pressure. This process was repeated three times at the beginning of every experiment, which took approximately 5–8 min. The atmosphere was then evacuated, purged with CO_2_, and evacuated again after each sampling to maintain Mars-relevant atmospheric conditions. Pressures could increase up to 1 inch Hg (34 mbar) in 1 week, after which the chamber was again evacuated to the required pressure. The cause of the increase was unclear, but media vaporization, degassing and leakage of the chamber are possible factors. Two Sun Blaster T5 high output fluorescent grow lamps were placed on the top of the tempered glass lid at a distance that allowed 62–70 μmol m^–2^ s^–1^ of continuous light exposure to the cultures. The range of 62–70 μmol m^–2^ s^–1^ was based on previous experiments by Harrold et al. (2018). The distance for proper light exposure was established by placing the handheld digital lux meter (URCERI) on the bottom of the chamber with the lid on. From the bottom of the chamber, all measurements were between 62 and 70 μmol m^–2^ s^–1^ with an accuracy of ±3% based on the meter manufacturer’s instructions. Each culture was set up in duplicate with abiotic controls that contained only media without cultures. To prevent sedimentation of algae and to allow a homogenized distribution of gasses and nutrients within the medium, the chamber was shaken at a rate of 150 revolutions per minute (RPM) on a standard shaker plate (VWR).

### Experimental Setup

Prior to initiating the low-pressure growth experiments, each culture was first inoculated on a solid agar plate (2% agar in respective media) using the streak plate method. An individual colony for each species was then picked from the agar plates and grown in its respective liquid medium under optimum conditions. The liquid cultures were used for all further experiments.

The growth phase of each culture under optimum conditions was tracked to the mid-logarithmic phase by measuring the optical density (OD) of a 1 ml sample of each culture using a GENESYS 10S UV-VIS spectrophotometer (Thermo Scientific) at 750 nm (OD_750_). The 750 nm wavelength is out of the absorbance range of algal pigments and hence is a preferred choice for OD measurement (Griffiths et al., 2011). The logarithmically growing cultures were then used to inoculate the algae growth experiments at the first set of low pressure conditions at 670 mbar.

For each experiment, 100 ml of autoclave-sterilized medium specific to each alga culture was used in 200 ml Erlenmeyer glass flasks. The flasks were first acid washed (10% nitric acid) and then rinsed three times with 18 MΩ cm^–1^ H_2_O. All equipment used for experimental setup and sampling was autoclaved before use. Cultures were inoculated with 10% inoculum in each case. The first sampling was performed immediately after inoculation to determine the initial optical density (OD) and cell counts of the inoculated culture. As soon as cultures were inoculated and sampled, they were placed in the low-pressure chamber, which was then evacuated and purged three times as described above. The temperature was maintained at 4.0 ± 0.1°C for the snow algae and room temperature (20.8 ± 2.6°C) for other algae. However, at the lowest pressure (80 mbar), algae other than snow algae were kept at 10.0 ± 0.1°C to reduce the vapor pressure under these low pressure conditions.

Algae growth was measured at four different pressures: 670, 330, 160, and 80 mbar. To allow cultures to potentially adapt to decreasing pressures, the inoculum for pressure conditions 330, 160, and 80 mbar was prepared so that half the volume of the inoculum was from cultures growing logarithmically outside the low-pressure growth chamber under normal atmospheric conditions, and the other half was an equal volume of the culture growing at 670, 330, and 160 mbar, respectively, after steady state was achieved.

### Sampling

Sampling was performed once a week for the duration of the experiments (33–62 days) to allow the cultures to reach the stationary growth phase. Algae growth was qualitatively determined by taking the OD_750_ measurements for each duplicate culture (*n* = 2) (Moheimani et al., 2013), whereas the quantitative growth measurements via cell counts were performed using two measurements each of duplicate cultures, making the total number of measurements *n* = 4. At all pressures, for growth experiments of the cultures for which cell counts were performed (*C. brevispina, D. salina* and *C. vulgaris*), cell concentrations for the last 2–4 time points were averaged and used to estimate the carrying capacity for all conditions except for *D. salina* at 670 mbar which was assumed to be at or near stationary growth at the end of the experiment (Supplementary Tables 1–3). For sampling, the low-pressure chamber was first brought to atmospheric pressure by opening the valve of the pressure outlet on the low-pressure chamber allowing air to enter the chamber. The cultures were then removed for sampling. Sampling was performed in a laminar flow hood (Horizon, LABCONCO) using aseptic techniques and took about 5–20 min. Samples were then returned to the low-pressure chamber, which was evacuated and purged as described above.

The laminar flow hood workspace was sterilized before and after sampling via UV lights (Pure UV) for 15 min and 70% ethanol to prevent contamination. Before sampling, cultures were first homogenized by gentle swirling to ensure uniform distribution of cells and then 1 ml of sample was extracted from each flask for growth measurement using a sterile pipet. Growth was measured immediately after sampling using OD_750_ for all cultures and cell count measurements for *C. brevispina, D. salina*, and *C. vulgaris* (Table 1). OD_750_ and cell counts measurements were used because both are a direct reflection of the biomass in each culture (Chioccioli et al., 2014).

### Growth Measurements

Algal cell counts were measured as previously described by Harrold et al. (2018) and Phillips-Lander et al. (2020). Briefly, samples were first vortexed to homogenize the cultures and 10 μL of the sample was used for each cell count reading using disposable Incyto C-chip hemocytometer chambers (Model #DHC-N01). For low-moderate cell concentrations, cells were counted within each of five large grid zones (V_*grid,L*_ = 1 × 10^–4^ ml), and for high cell concentrations, cells were counted within five to thirteen small grid zones (V_*grid*,sm_ = 1 × 10^–6^). For the lowest cell concentrations, an entire hemocytometer grid was counted. All cell counts were performed using an Olympus BH microscope under 400× magnification. Concentrations of algal cells were determined according to Eq. (1):


(1) $${\text{C}}_{\text{algae}}=\frac{\text{N}}{\text{n}\times {\text{V}}_{\text{grid}}}$$

where algae cell concentration (*C*_*algae*_) (cells ml ^–1^) was determined by measuring the total number of cells (*N*) in the grid blocks (*n*), where *V*_*grid*_ is the volume per grid used for enumeration. All cell counts were performed using two measurements of duplicate cultures, making the total number of measurements *n* = 4, and the standard deviation was calculated using the Excel Analysis ToolPak function (Excel, Microsoft Office 365, v. 16.43).

### Modeling Cell Growth

To determine whether culture growth was statistically significant, *P*-values and *R*^2^ (correlation coefficients) were calculated, with values of *P* < 0.05 considered statistically significant. The goodness of fit determined by *R*^2^ (Kuśnierz and Łomotowski, 2015) was measured for the regression analysis of each growth curve of the algae cultures plotted as a log scale of their exponential growth measured as average cell counts against time at each pressure condition (Supplementary Tables 9, 10). The statistical analysis was performed in Microsoft Excel Analysis Tool Pak (v. 16.43) (Supplementary Table 9).

The primary, overarching goal of this work was to test growth under low pressures, and we therefore performed fewer sampling sessions to minimize the amount of time that cultures were returned to normal terrestrial atmospheric conditions during sampling. These growth curves therefore contain fewer time points than many microbial growth experiments, and we anticipated that fitting these data with the logistic growth curve (Eq. 2) would be less constrained than had we more data points. However, fitting these data with the logistic growth curve (Eq. 2) can help assess the potential of algae for production of oxygen and food for astronauts by helping constrain the doubling time and lag phase duration (LPD) of these cultures under low pressure conditions. Although the generated curves are less constrained, we did fit the data using the logistic growth curve using the Solver function in Microsoft Excel (v. 16.43). The logistic growth curve fittings are in Supplementary Figures 1–24.

The logistical growth equation (Eq. 2) was used to fit algal cell concentration data from 0–62 days of incubation, where the average of the two cell count measurements of each culture (*n* = 2) was fit separately (Supplementary Figures 1–24).


(2) $${\text{C}}_{\text{algae}}\left(\text{t}\right)=\frac{{\text{C}}_{\mathrm{algae},\max}}{1+{\text{e}}^{-r\left(t-{\text{t}}_{\text{half}}\right)}}$$

where *C_*algae*_ (t)* is the concentration of algae at time *t*, *C_*algae*_,_*max*_* is the maximum concentration of algae or the carrying capacity of the culture, *t-half* is the time at the sigmoid midpoint, and *r* is the slope at the sigmoid midpoint. *C_*algae*_,_*max*_* was estimated by averaging the last 2–4 cell concentration measurements except for *D. salina* at 670 mbar, for which 1 point was used (Supplementary Tables 1–3 and Table 2), and was then used as an input in fitting the logistic growth curve to the data using the Excel Solver function in the Analysis Tool Pak (v. 16.43). Logistical growth curves were fit by minimizing the residual sum of squares and yielding best-fit *t-half* and *r* values, where the mean of cell counts (Y), Standard error (SE) of (Y), Sum of Square of Residuals, Critical T, Degree of freedom and Confidence intervals were generated and were then used by the Solver function in the Excel for Best-fit logistic curve fitting (Supplementary Tables 4–6; Motulsky and Christopoulos, 2004). To examine differences in the time required by each species to acclimatize at different pressures, the length of the lag phase was estimated as the point at which the algae concentration calculated using the logistic growth curve was 15% that of the carrying capacity. This allowed a comparison of the duration of the estimated lag phase between different species, and between the same species at different pressures.

**TABLE 2:** Table showing the carrying capacities (*C_*algae*_,_ max_)* computed at different pressures for *Chlorella vulgaris* (CV) *Dunaliella salina (DS)*, and *Chloromonas.*

Pressure (mbar)	Average carrying capacity of duplicates (C_*algae*_,_max_)*	Uncertainty*	n (number of time points the average carrying capacity is based on)
***Chloromonas brevispina***			

670 ± 20	161.1 × 10^4^	5.2 × 10^4^	2
330 ± 20	198.0 × 10^4^	8.8 × 10^4^	3
160 ± 20	86.8 × 10^4^	6.2 × 10^4^	3
80 ± 2.5	43.4 × 10^4^	2.5 × 10^4^	3

***Dunaliella salina***			

670 ± 20	2.3 × 10^6^	1.5 × 10^5**^	1
330 ± 20	121.3 × 10^4^	7.5 × 10^4^	3
160 ± 20	30.0 × 10^5^	4.6 × 10^5^	4
80 ± 2.5	15.8 × 10^4^	1.3 × 10^4^	4

***Chlorella vulgaris***			

670 ± 20	32.8 × 10^4^	1.1 × 10^4^	3
330 ± 20	78.8 × 10^4^	3.6 × 10^4^	3
160 ± 20	13.0 × 10^5^	1.5 × 10^5^	4
80 ± 2.5	57.1 × 10^4^	4.5 × 10^4^	3

*^∗^The carrying capacities are the averages of the duplicate experiments reported in Supplementary Table 11 with the uncertainties propagated for the average of the duplicate experiments.*

*^∗∗^This uncertainty represents half the range between the duplicates.*

The exponential growth rate equation (Eq. 3) was used to fit algal concentration data spanning 0 days of incubation up to one time point beyond the best fit T_*half*_ value as determined from the logistic curve, where the average of the duplicate measurements of each culture (*n* = 2) was fit separately (Table 3):


(3) $${\text{C}}_{\text{algae}}\left(\text{t}\right)={\text{C}}_{\mathrm{algae},0}{\text{e}}^{\text{rt}}$$

**TABLE 3:** Estimated Lag Phase Duration (LPD), growth rate (r), doubling time (T_d_), and correlation coefficient (*R*^2^) value for the candidate algae at different pressures.

Pressure (mbar)		*Chloromonas brevispina* (CB)					*Dunaliella salina* (DS)					*Chlorella vulgaris* (CV)			
	^a^LPD*	^b^r*	^c^T_d_*	^d^ *R* ^2^		^a^LPD	^b^r	^c^T_d_	^d^R^2^		^a^LPD	^b^r	^*c*^ T_d_	^d^R^2^	
				^CB1^	^CB2^				^CB1^	^CB2^				^CB1^	^CB2^
670 ± 20	6.8 ± 0.2	0.11 ± 0.02	6.4 ± 1.3	0.86	0.91	25.8 ± 1.4	0.09 ± 0.01	8.2 ± 1.4	0.73	0.87	8.61 ± 0.07	0.02 ± 0.005	32.5 ± 7.4	1	0.89
330 ± 20	10.7 ± 0.7	0.13 ± 0.02	5.4 ± 1.1	0.89	0.86	9.7 ± 0.7	0.19 ± 0.03	3.7 ± 0.7	0.92	0.99	2.6 ± 0.1	0.13 ± 0.03	5.7 ± 1.6	0.89	0.89
160 ± 20	9.0 ± 0.4	0.08 ± 0.01	9.0 ± 1.7	0.93	0.86	2.7 ± 0.8	0.07 ± 0.02	9.5 ± 3.6	0.81	0.91	0.20 ± 0.05	0.14 ± 0.06	3.9 ± 2.0	0.78	0.79
80 ± 2.5	13.6 ± 0.2	0.11 ± 0.02	6.1 ± 1.1	0.94	0.83	0.8 ± 0.3	0.08 ± 0.03	8.6 ± 3.3	0.84	0.69	6.6 ± 2.1	0.11 ± 0.04	6.4 ± 2.6	0.89	0.78

*^∗^Lag phase duration (LPD), growth rate (r), doubling time (T_d_), for Chloromonas brevispina (CB), Dunaliella salina (DS), and Chlorella vulgaris (CV) were the averages of the duplicate experiments reported in Supplementary Table 12 with the uncertainties on LPD, doubling time and growth rate are propagated for the average of the duplicate experiments.*

*^a^Lag phase duration (LPD) was estimated as 15% of the culture carrying capacity and is reported in days.*

*^b^Growth rate (r) as solved using the exponential growth equation (Eq. 3) and is reported as per day.*

*^c^Doubling time (T_d_) is the time it takes for a population to double in size found using Eq. 4 and is reported in days.*

*^d^Correlation coefficient (R^2^) is used to measure the goodness of fit for non-linear regression.*

where *C_*algae*_ (t)* is the concentration of algae at time *t*, *C*_*algae*,0_ is the initial algal concentration in the experiment resulting from inoculation before growth has occurred and is input as a fixed parameter based on measured values, and the equation was solved for the growth rate *(r)* using Microsoft Excel. The growth rate (*r*) was then used to determine the doubling time (*T*_*d*_) using Eq. 4:


(4) $${\text{T}}_{\text{d}}=\frac{\ln\left(2\right)}{\text{r}}$$

The goodness of fit (*R*^2^) for the exponential algal growth models of each culture condition is given in Table 3.

## Results

### Algae Growth Dynamics Under Different Pressure Conditions

**FIGURE 2:**
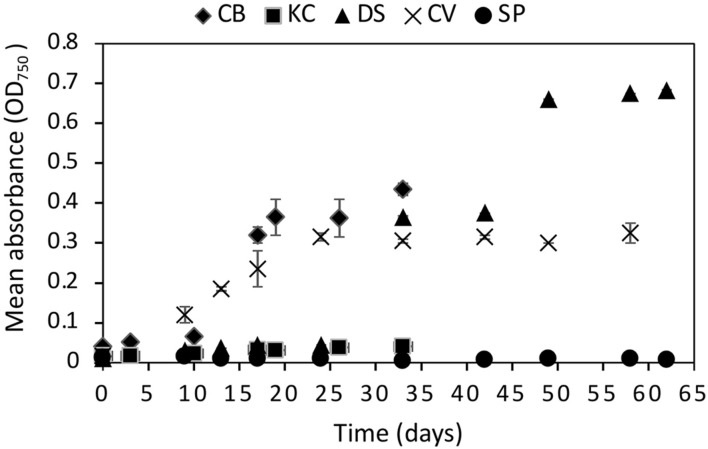
Growth curves of all five algae *Chloromonas brevispina* (CB), *Kremastochrysopsis austriaca* (KC), *Chlorella vulgaris* (CV), Spirulina plantensis (SP) and *Dunaliella salina* (DS) grown at low pressures of 670 ± 20 mbar plotted as a mean of absorbance measured by optical density measurement at 750 nm. Error bars represent the range in OD_750_ values between duplicate experiments. Where error bars are not visible, they fall within the symbol.

The statistical analysis on the OD measurements at 670 mbar indicated that *C. brevispina, C. vulgaris, K. austriaca*, and *D. salina* showed statistically significant (*p* < 0.05) growth at 670 mbar, whereas *S. plantensis* did not show statistically significant growth (Figure 2 and Supplementary Table 9). However, despite showing statistically significant growth at 670 mbar, *K. austriaca* showed very minimal growth at this pressure (OD values slightly more than doubled over the course of the experiment). We therefore chose the strains *C. brevispina, D. salina*, and *C. vulgaris* as candidate strains for further detailed quantitative growth analysis using cell counts at lower pressures (Supplementary Tables 1–3 and Figure 2).

*C. brevispina*, *D. salina*, and *C. vulgaris* exhibited exponential growth at 670 mbar as indicated by the goodness of fit (*R*^2^) values obtained by the exponential growth models of their OD values which measured as 0.93, 0.90, and 0.88, respectively (Supplementary Figure 25 and Supplementary Table 9).

#### Carrying Capacity

The carrying capacity for the cultures, measured as the average of the last *n* = 2–4 time points except for *D. salina* at 670 mbar, for which 1 point was used, ranged from 16.0 ± 1.3 × 10^4^ cells/ml to 30.0 ± 4.6 × 10^5^ cells/ml. The highest carrying capacity for each species was observed at the pressures of 330 mbar for *C. brevispina* at 19.8 ± 0.9 × 10^5^ cells/ml, at 160 mbar for *D. salina* at 30.0 ± 5.6 × 10^5^ cells/ml and for *C. vulgaris* at 13.0 ± 1.5 × 10^5^ cells/ml (Table 2).

#### Doubling Time

The doubling time for the cultures ranged from 3.7 ± 0.7 to 32.5 ± 7.4 days, with the fastest doubling time for each species being at the pressures of 330 mbar for *C. brevispina* (5.4 ± 1.1 days) and *D. salina* (3.7 ± 0.7 days) and at 160 mbar for *C. vulgaris* (3.9 ± 2.0 days). Due to our experimental setup, designed to minimize changes in pressure required by sampling, and thus with measurements 1 week apart, the uncertainty on the doubling times is large (Table 3), but these fastest doubling times under low pressures are similar to those previously measured for *C. brevispina* under optimum conditions (5.2 ± 0.1 days; Harrold et al., 2018).

#### Lag Phase Duration

The estimated LPD for the cultures ranged from 0.20 ± 0.05 to 25.8 ± 1.4 days, with the shortest lag phase for each species being at the pressures of 670 mbar for *C. brevispina* (6.8 ± 0.2 days), at 80 mbar for *D. salina* (0.8 ± 0.3 days*)*, and at 160 mbar for *C. vulgaris* (0.20 ± 0.05 days) (Table 3).

#### Trends With Decreasing Pressure

**FIGURE 3:**
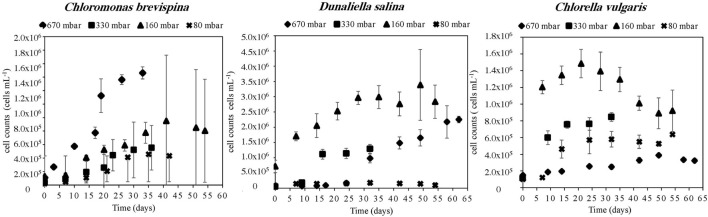
Growth curves of *Chloromonas brevispina* (CB), *Chlorella vulgaris* (CV), and *Dunaliella salina* (DS) at pressures of 670 ± 20 mbar, 330 ± 20 mbar, 160 ± 20 mbar, and 80 ± 2.5 mbar plotted as a mean value (*n* = 4) of duplicate cell count measurements of duplicate experiments. Error bars are 1 standard deviation of mean cell count values, and all data are shown in Supplementary Tables 1–3. Where error bars are not shown they lie within the points.

The strain *C. vulgaris* showed the clearest trends with decreasing pressure. With the exception of the 80 mbar pressure, *C. vulgaris* displayed increasing carrying capacities with decreasing pressure, with carrying capacities reaching 32.8 ± 1.1 × 10^4^ cells/ml at 670 mbar, 78.8 ± 3.6 × 10^4^ cells/ml at 330 mbar, and 13.0 ± 1.5 × 10^5^ cells/ml at 160 mbar (Table 2). Similarly, with the exception of the 80 mbar pressure condition, a decreasing trend was observed in the estimated LPD with decreasing pressure, with the length of the estimated lag phase decreasing from 8.61 ± 0.07 at 670 mbar to 2.6 ± 0.1 at 330 mbar to 0.20 ± 0.05 at 160 mbar (Tables 2, 3 and Figure 3).

Similarly, with decreasing pressure, *D. salina* showed a consistent decrease in the estimated LPD from 25.8 ± 1.4 days at 670 mbar, to 9.7 ± 0.7 days at 330 mbar, to 2.7 ± 0.8 days at 160 mbar, and 0.8 ± 0.3 days at 80 mbar. However, no clear trend in carrying capacity was observed for *D. salina*, with the highest carrying capacity of 30.0 ± 4.6 × 10^5^ cells/ml observed at 160 mbar pressure (Tables 2, 3 and Figure 3). For *C. brevispina*, LPD generally increased with decreasing pressure, and no clear trend was observed for carrying capacity with pressure (Tables 2, 3 and Figure 3).

## Discussion

Human exploration of Mars is one of the key scientific and technological undertakings of our time. Current research is ongoing to successfully support astronauts’ food and oxygen needs for long-term space exploration journeys including to Mars (Massa et al., 2017). The results of this research underscore the critical need for advanced BLSS to support human life during extended space flight and on long planetary surface expeditions (Monje et al., 2003; Revellame et al., 2021). Algae are considered an excellent food source for astronauts because they (1) contain all the essential amino acids, (2) are more digestible than traditional plant protein and (3) grow faster than traditional crops (wheat, rice, corn, etc.) (Bleakley and Hayes, 2017; Koyande et al., 2019; Yang et al., 2019). Algal oil also contains substantial contents of poly-unsaturated fatty acid (PUFA) and algal-specific super-antioxidants, both of which may have beneficial effects for astronauts exposed to harsh space environments (Harwood, 2019; Yang et al., 2019). Recent results also signified the biomedical applications of astaxanthin, a pigment produced by algae, in preventing certain cancers, aging, macular degeneration, and inflammation (Grimmig et al., 2017). All these reports suggests that algae can be a competitive food option for food and oxygen production in long-term space exploratory missions (Yang et al., 2019).

However, optimization of algal growth for their use in self-sustaining BLSS is needed. Therefore, the goal of this study was to grow algae for potential oxygen and food production under low pressure conditions, such as might be possible in an enclosed low-pressure environment made with flexible materials on Mars. Flexible materials, such as those used for the extravehicular mobility unit (EMU) spacesuit that enables pressurized oxygen, ventilation, as well as carbon dioxide, water vapor, and trace contaminant removal, maintains a pressure of 296 mbar (4.3 psi) (National Research Council, 1997). Low pressure (∼200–300 mbar) martian or lunar greenhouses and inflatable structures have already been conceptualized and designed (e.g., Cadogan et al., 1999; Wheeler and Martin-Brennan, 2000). The use of flexible materials to make inflatable structures would considerably reduce the mass and volume of any martian greenhouse structure making it more viable for deployment. Therefore, the ability to grow photosynthetic organisms under low pressures (<296 mbar) facilitates the use of BLSS that could be utilized on Mars. Furthermore, pressurizing such a structure initially with the martian atmosphere would reduce transported oxygen/air resource requirements.

In this study, the maximum growth for each species was observed at the pressures of 330 mbar for *C. brevispina* with 19.8 ± 0.9 × 10^5^ cells/ml and at 160 mbar for *D. salina* with 30.0 ± 5.6 × 10^5^ cells/ml and for *C. vulgaris* with 13.0 ± 1.5 × 10^5^ cells/ml. To put these cell concentrations into context, here we compare them with optimum growth conditions as reported in both natural and laboratory conditions. For *C. brevispina*, under optimum conditions, cell concentrations were observed reaching 10^6^ cells/ml (Hoham et al., 1979; Harrold et al., 2018), and for *D. salina*, the highest cell concentrations reached 10^6^ –10^7^ cells/ml under laboratory conditions (García et al., 2006; Hadi et al., 2008; Ahmed et al., 2017). For *C. vulgaris*, the maximum growth under optimum conditions was also observed to range from 10^6^ to 10^7^ cells/ml under laboratory conditions (Mandalam and Palsson, 1995; Adamczyk et al., 2016; Sánchez-Saavedra et al., 2020). The ability of our candidate algae species to grow under low pressure conditions and reach cell concentrations close to maximum cell counts observed for these species under optimum conditions, makes them excellent candidates to be used for BLSS.

The results of our experiments also show that three species showed substantial growth at 80 mbar and 160 mbar (Supplementary Figures 27, 28), well below the 200–300 mbar lower limit generally proposed for flexible materials on Mars, and well below the value thought to be the limit for vascular plant growth (Hublitz et al., 2004; Paul and Ferl, 2006; Richards et al., 2006). Under these conditions of very low pressure (80 mbar), the growth rates of the cultures *C. brevispina, D. salina*, and *C. vulgaris* were relatively slow (with doubling times of ∼5–9 days, although these are comparable to *C. brevispina* growth under optimum conditions (Harrold et al., 2018).

According to previous estimates, each astronaut performing 2 h of intense physical activity each day would consume approximately 1 kg of O_2_ per day (Horneck et al., 2003), which can be photosynthetically produced by bio-fixation of 1.3 kg of CO_2_ (Verseux et al., 2016). Previous work indicates that for C. *vulgaris* species, a maximal bio-fixation rate of 1.4 g CO_2_ /L/d was observed at a cell concentration of 1.3 × 10^7^ cells/ml under optimum conditions (Adamczyk et al., 2016) and for D. salina species, under optimum conditions, the CO_2_ bio-fixation rate was observed to range from 0.71 g CO_2_/L/d to 1.102 g CO_2_/L/d at maximum biomass concentrations (Mortezaeikia et al., 2016). Based on these values of CO_2_ bio-fixation rates, it can be estimated that the cell counts of *C. vulgaris* and *D. salina* measured in our experiments reaching 13.0 ± 1.5 × 10^5^ cells/ml and 30.0 ± 4.6 × 10^5^ cells/ml at 160 mbar, respectively, could potentially generate enough oxygen for astronaut consumption. Snow algae *C. brevispina* is also known to be an important CO_2_ sink in snow environments (Williams et al., 2003) and their cell counts reaching 19.8 ± 0.9 × 10^5^ cells/ml at 330 mbar in our experiments indicate their potential to photosynthetically generate substantial oxygen via CO_2_ bio-fixation. These calculations, however, are simply estimates as the photosynthesis machinery of algae can be influenced by various environmental factors [light exposure, pressure, activity of reactive oxygen species (ROS), pH fluctuations, etc.]. Therefore, even though the biomass data suggest that these strains could be pursued as food and oxygen producers on Mars, further research is needed to directly optimize and quantify CO_2_ fixation and O_2_ generation under these low-pressure settings.

The observed decrease in duration of the estimated lag phase with decreasing pressure for *D. salina* and *C. vulgaris*, and the increasing carrying capacities with decreasing pressure observed for *C. vulgaris* (Table 2) suggest that the cultures may be acclimatizing to the decreasing pressure conditions. Few studies have explored the mechanisms of adaptation of microorganisms under low pressure conditions (Kanervo et al., 2005; Nicholson et al., 2013; Murukesan et al., 2016; Schuerger and Nicholson, 2016; Verseux, 2020). Previous research on prokaryotes growing at low pressures, including transcription analysis, revealed that owing to their specialized adaptations to thrive in extreme environments, extremophiles are most likely to be better suited to survive under low pressures conditions (Schuerger et al., 2013; Verseux, 2020). This could explain the successful growth at low pressure of the extremophilic candidate strain *D. salina* and *C. brevispina*, where their natural adaptations to cope with high salinity, cold temperatures, and high irradiance might be responsible for their tolerance to low pressures as well. Some reports also suggest that microorganisms could evolve toward higher tolerance if exposed to low pressure over multiple generations (Verseux, 2020). This may explain the trend observed in the *C. vulgaris* species where the increasing trend in carrying capacity was observed with decreasing pressure. Additionally, *C. vulgaris* is globally distributed in both aquatic and terrestrial habitats (Bock et al., 2011; Aigner et al., 2020) suggesting that the species has adaptations to survive in these contrasting habitats. The molecular mechanisms of such adaptations are not clearly understood (Aigner et al., 2020) but may also contribute to the tolerance to low pressures as well.

Further analysis of the molecular basis of low-pressure adaptations will be required to understand different growth dynamics of algae species under low pressure conditions and may reveal key genes or quantitative trait loci that are involved in growth at low pressure. Such key genes or quantitative trait loci could potentially be selected for use in breeding studies, resulting in more useful algal strains. In addition, long-term growth under low pressure on Earth should lead to the development of strains with elevated productivities under low pressure. Together, such studies would accelerate the development of an algal-based BLSS for Mars.

## Conclusion

Life support represents one of the most critical technologies needed for successful and safe long-term deep space human exploration missions and will require substantial amounts of both oxygen and food production. The results from this study contribute to the development of a BLSS by demonstrating the potential contributions of three candidate species *C. brevispina, D. salina*, and *C. vulgaris.* All three of the candidates showed exponential growth at low pressures of 80 mbar and 160 mbar, which indicates the possibility of using inflatable greenhouses to produce oxygen on the surface of Mars. If these cultures produce approximately similar O_2_ yields per unit of dry biomass as recorded previously (Kirensky et al., 1968; Gitelson, 1992), the biomass of the algae used as food could also generate enough O_2_ for the astronauts’ use. In addition, the lag phases of *D. salina* and *C. vulgaris* decreased with decreasing pressure, and the carrying capacity of *C. vulgaris* increased with decreasing pressure, which suggests that the cultures may be acclimatizing to the decreasing pressure conditions and may be increasingly useful in BLSS. Together these results indicate that these species may be able to contribute to potential BLSS on Mars using low pressure (∼200–300 mbar) greenhouses and inflatable structures that have already been conceptualized and designed.

## Data Availability Statement

The data supporting the conclusions of this paper are available in the Supplementary Material.

## Author Contributions

LC, EH, DM, and WR contributed to the conception and design of the study. LC conducted the experiments. EH and LC analyzed the data and performed the statistical analysis. CA contributed to the experimental design and wrote a section of the manuscript. JR and DR contributed to sample collection and experimental setup. All authors contributed to manuscript revision, read, and approved the submitted version.

## Conflict of Interest

The authors declare that the research was conducted in the absence of any commercial or financial relationships that could be construed as a potential conflict of interest.

## Publisher’s Note

All claims expressed in this article are solely those of the authors and do not necessarily represent those of their affiliated organizations, or those of the publisher, the editors and the reviewers. Any product that may be evaluated in this article, or claim that may be made by its manufacturer, is not guaranteed or endorsed by the publisher.
