# Supplementary material for: Investigating the Growth of Algae Under Low Atmospheric Pressures for Potential Food and Oxygen Production on Mars
Source: Front Microbiol. 2021 Nov 12;12:733244. doi: 10.3389/fmicb.2021.733244 (PMC8633435; doi:10.3389/fmicb.2021.733244)
Supplement: Supplementary file 1 [file Data_Sheet_1.docx]

**Supplementary Information for:**

**Investigating the growth of algae under low atmospheric pressures for potential food and oxygen production on Mars**

L. M. Cycil^1^, E.M. Hausrath^1^, D. W. Ming^2^, C. Adcock^1^, J. Raymond^3^, D. Remias^4^, W. Ruemmele^2^

^1^UNLV, Department of Geoscience, Las Vegas, NV 89154, ^2^NASA JSC, Houston, TX 77058. ^3^UNLV School of Life Sciences, Las Vegas, NV 89154, ^4^School of Engineering, University of Applied Sciences Upper Austria, Wels, Austria.

| **Table of contents** | | |
| --- | --- | --- |
| Supplementary Tables for cell count measurements | Page no. 3-7 | - Table S1- S3. Cell counts, mean and Standard deviation measured for duplicate cultures of *Chloromonas brevispina* (CB), *Dunaliella salina* (DS) and *Chlorella vulgaris* (CV) measured as cells per ml. - Table S4-S6. Best-fit logistical model parameters for *Chloromonas brevispina*, *Dunaliella salina (DS), Chlorella vulgaris (CV*) growth used for curve fitting with the Solver function in Microsoft Excel (v. 16.43). |
| Supplementary plots for logistic growth curve fittings | Page no. 8-19 | - Figure S1-S8. Best-fit logistic curve fitting for duplicates of *Chloromonas brevispina (CB1 &CB2)* cell/ml at 670 mbar, 330 mbar, 160 mbar and 80 mbar. - Figure S9-16. Best-fit logistic curve fitting for duplicates *Dunaliella salina (DS1 & DS2) cell*/ml at 670 mbar, 330 mbar, 160 mbar and 80 mbar. - Figure S17-24. Best-fit logistic curve fitting for duplicates *Chlorella vulgaris (CV2 & CV2)* cell/ml at 670 mbar, 330 mbar, 160 mbar and 80 mbar. |
| Supplementary tables for Optical Density measurements | Page no.  19-21 | - Table S7. Optical density (OD) and Mean (Avg) measured for duplicate cultures of *Chloromonas brevispina* (CB) and *Kremastochrysopsis austriaca (KC).* - Table S8. Optical density (OD) and Mean (Avg) measured for duplicate cultures of *Chlorella vulgaris* (CV), *Dunaliella salina (DS)* and *Spirulina plantensis (SP).* |
| Supplementary Data for statistical analysis | Page no.  21-26 | - Table S9: P-value and R^2^ calculated from the growth of C*hloromonas brevispina (CB), Kremastochrysopsis austriaca, (KC)., Dunaliella salina (DS), Chlorella vulgaris (CV)* and *Spirulina plantensis (SP*), measured at 670 mbar plotted as log scale values of OD measurements against the time (days) using Microsoft Excel Analysis Toolkit. - Figure S25: Best-fit exponential models fit to duplicate algae growing at 670 mbar data spanning from day 0 to one data point past T_half_. - Table S10: R^2^ calculated from the Best-fit exponential growth model of C*hloromonas brevispina (CB), Dunaliella salina (DS)* and *Chlorella vulgaris (CV)* cultures measured at different pressures plotted as log scale values of cell counts/ ml measurements against the time (days) using Microsoft Excel Analysis Toolkit. - Figure S26: A) Modified low pressure chamber, held 670 mbar pressure for 36 hours, after which, it was pumped down to the required pressure. It could not hold it at 330 mbar or lower pressures. B) Aluminum Vacuum Chamber (Slickvacseal) used for algae growth experiments at 330 mbar, 160 mbar and 80 mbar and was able to hold the low pressures for a week. - Table S11. Table showing the carrying capacities (*C_algae_, _max_)* computed at different pressures as the average carrying capacities for duplicates (1, 2) of algae *Chlorella vulgaris* (CV) *Dunaliella salina (DS)* and *Chloromonas brevispina* (*CB*). - Table S12. Best-fit exponential model parameters computed for the duplicate cultures of candidate algae at different pressures. - Figure S27. Comparison of growth curves of *Chloromonas brevispina (CB), Chlorella vulgaris (CV)* and *Dunaliella salina (DS)* at 80 ± 2.5 mbar plotted as a mean value (n=4) of cell count measurements per ml. Error bars are the standard deviation of mean cell counts values. - Figure S28. Comparison of growth curves of *Chloromonas brevispina (CB), Chlorella vulgaris (CV)* and *Dunaliella salina (DS)* at 160 ± 20 mbar plotted as a mean value (n=4) of cell count measurements per ml. Error bars are the standard deviation of mean cell counts values. Larger variations in later time points might be because some cultures are still growing, and some reached death phase. |

**Supplementary Tables for cell counts**

Table S1. Cell counts, mean and standard deviation measured for duplicate cultures of *Chloromonas brevispina* (CB) measured as cells per ml.

| **Pressure 670 mbar (±20mbar)** | | |  |  |  |  |
| --- | --- | --- | --- | --- | --- | --- |
| **Incubation days** | **CB1(1)** | **CB1(2)** | **CB2(1)** | **CB2(2)** | **Average** | **Standard Deviation** |
| 0 | 1.111E+05 | 9.999E+04 | 1.111E+05 | 1.189E+05 | 1.103E+05 | 7.8E+03 |
| 3 | 2.01E+05 | 3.34E+05 | 2.67E+05 | 2.67E+05 | 2.67E+05 | 3.4E+04 |
| 10 | 6.00E+05 | 5.50E+05 | 6.00E+05 | 5.50E+05 | 5.75E+05 | 2.4E+04 |
| 17 | 8.00E+05 | 6.50E+05 | 8.00E+05 | 8.50E+05 | 7.75E+05 | 8.5E+04 |
| 19 | 1.20E+06 | 1.30E+06 | 1.10E+06 | 1.70E+06 | 1.33E+06 | 2.5E+05 |
| 26 | 1.450E+06 | 1.700E+06 | 1.550E+06 | 1.550E+06 | 1.563E+06 | 7.3E+04 |
| 33 | 1.500E+06 | 1.800E+06 | 1.600E+06 | 1.750E+06 | 1.663E+06 | 8.9E+04 |
| **Pressure 330 mbar (±20mbar)** | | |  |  |  |  |
| **Incubation days** | **CB1(1)** | **CB1(2)** | **CB2(1)** | **CB2(2)** | **Average** | **Standard Deviation** |
| 0 | 1.27E+05 | 1.11E+05 | 2.00E+05 | 1.29E+05 | 1.42E+05 | 4.0E+04 |
| 7 | 1.27E+05 | 1.33E+05 | 1.86E+05 | 1.28E+05 | 1.43E+05 | 2.8E+04 |
| 14 | 1.0E+06 | 7.5E+05 | 1.0E+06 | 7.0E+05 | 8.6E+05 | 1.6E+05 |
| 20 | 1.60E+06 | 1.40E+06 | 1.60E+06 | 1.05E+06 | 1.41E+06 | 2.6E+05 |
| 23 | 2.15E+06 | 2.20E+06 | 1.80E+06 | 1.75E+06 | 1.98E+06 | 2.3E+05 |
| 30 | 2.30E+06 | 1.60E+06 | 2.15E+06 | 1.45E+06 | 1.88E+06 | 4.1E+05 |
| 36 | 1.85E+06 | 2.55E+06 | 1.85E+06 | 2.10E+06 | 2.09E+06 | 3.3E+05 |
| **Pressure 160 mbar (±20mbar)** | | | | | | |
| **Incubation days** | **CB1(1)** | **CB1(2)** | **CB2(1)** | **CB2(2)** | **Average** | **Standard Deviation** |
| 0 | 1.267E+05 | 1.333E+05 | 1.333E+05 | 1.278E+05 | 1.303E+05 | 3.6E+03 |
| 7 | 1.20E+05 | 1.80E+05 | 1.50E+05 | 1.30E+05 | 1.45E+05 | 2.6E+04 |
| 14 | 3.80E+05 | 4.00E+05 | 3.90E+05 | 4.60E+05 | 4.08E+05 | 3.6E+04 |
| 20 | 5.50E+05 | 6.00E+05 | 5.00E+05 | 4.40E+05 | 5.23E+05 | 6.8E+04 |
| 27 | 5.00E+05 | 7.00E+05 | 5.50E+05 | 6.00E+05 | 5.88E+05 | 8.5E+04 |
| 34 | 9.0E+05 | 5.5E+05 | 8.0E+05 | 8.5E+05 | 7.8E+05 | 1.6E+05 |
| 41 | 8.5E+05 | 1.1E+06 | 1.0E+06 | 9.0E+05 | 9.5E+05 | 9.8E+05 |
| 51 | 8.0E+05 | 9.0E+05 | 8.1E+05 | 8.9E+05 | 8.5E+05 | 8.6E+05 |
| 54 | 9.7E+05 | 8.0E+05 | 7.0E+05 | 7.5E+05 | 8.1E+05 | 7.6E+05 |
| **Pressure 80 mbar (±2.5mbar)** | | | | | | |
| **Incubation days** | **CB1(1)** | **CB1(2)** | **CB2(1)** | **CB2(2)** | **Average** | **Standard Deviation** |
| 0 | 3.11E+04 | 1.78E+04 | 2.11E+04 | 1.11E+04 | 2.03E+04 | 8.3E+03 |
| 7 | 1.0E+05 | 5.8E+04 | 1.7E+04 | 1.0E+04 | 4.6E+04 | 4.2E+04 |
| 14 | 1.19E+05 | 7.78E+04 | 1.13E+05 | 1.22E+05 | 1.08E+05 | 2.1E+04 |
| 21 | 2.01E+05 | 2.32E+05 | 1.51E+05 | 2.44E+05 | 2.07E+05 | 4.2E+04 |
| 28 | 5.10E+05 | 3.40E+05 | 3.70E+05 | 4.30E+05 | 4.13E+05 | 7.5E+04 |
| 35 | 5.40E+05 | 4.70E+05 | 4.30E+05 | 3.90E+05 | 4.58E+05 | 6.4E+04 |
| 42 | 4.40E+05 | 4.10E+05 | 5.10E+05 | 3.70E+05 | 4.33E+05 | 5.9E+04 |

* CB1 and CB2 are the duplicate experiments, and (1) and (2) are the duplicate measurements of each individual culture.

Table S2. Cell counts, mean (Avg) and Standard deviation (Std Dev) measured for duplicate cultures of *Dunaliella salina* (DS) measured as cells per ml.

| **Pressure 670 mbar (±20mbar)** | | |  |  |  |  |
| --- | --- | --- | --- | --- | --- | --- |
| **Incubation days** | **DS1(1)** | **DS1(2)** | **DS2(1)** | **DS2(2)** | **Average** | **Standard Deviation** |
| 0 | 6.0E+04 | 8.8E+04 | 5.8E+04 | 7.6E+04 | 7.0E+04 | 1.4E+04 |
| 9 | 6.78E+04 | 5.67E+04 | 6.22E+04 | 7.67E+04 | 6.58E+04 | 8.5E+03 |
| 13 | 7.8E+04 | 6.8E+04 | 7.6E+04 | 1.00E+05 | 8.0E+04 | 1.4E+04 |
| 17 | 8.9E+04 | 7.4E+04 | 8.7E+04 | 1.11E+05 | 9.0E+04 | 1.5E+04 |
| 24 | 1.67E+05 | 1.89E+05 | 1.44E+05 | 1.51E+05 | 1.63E+05 | 2.0E+04 |
| 33 | 1.08E+06 | 9.8E+05 | 7.7E+05 | 1.07E+06 | 9.8E+05 | 1.4E+05 |
| 42 | 1.74E+06 | 1.53E+06 | 1.30E+06 | 1.36E+06 | 1.48E+06 | 2.0E+05 |
| 49 | 1.70E+06 | 1.97E+06 | 1.33E+06 | 1.60E+06 | 1.65E+06 | 2.6E+05 |
| 58 | 1.50E+06 | 2.20E+06 | 2.20E+06 | 2.80E+06 | 2.18E+06 | 5.3E+05 |
| 62 | 2.30E+06 | 2.35E+06 | 2.10E+06 | 2.25E+06 | 2.25E+06 | 1.1E+05 |
| **Pressure 330 mbar (±20mbar)** | | |  |  |  |  |
| **Incubation days** | **DS1(1)** | **DS1(2)** | **DS2(1)** | **DS2(2)** | **Average** | **Standard Deviation** |
| 0 | 5.67E+04 | 5.89E+04 | 4.89E+04 | 4.22E+04 | 5.17E+04 | 7.6E+03 |
| 9 | 1.00E+05 | 1.80E+05 | 2.00E+05 | 2.30E+05 | 1.78E+05 | 5.6E+04 |
| 16 | 1.00E+06 | 1.05E+06 | 1.05E+06 | 1.35E+06 | 1.11E+06 | 1.6E+05 |
| 24 | 1.20E+06 | 1.00E+06 | 1.00E+06 | 1.35E+06 | 1.14E+06 | 1.7E+05 |
| 33 | 1.25E+06 | 1.25E+06 | 1.15E+06 | 1.50E+06 | 1.29E+06 | 1.5E+05 |
| **Pressure 160 mbar (±20mbar)** | | |  |  |  |  |
| **Incubation days** | **DS1(1)** | **DS1(2)** | **DS2(1)** | **DS2(2)** | **Average** | **Standard Deviation** |
| 0 | 9.5E+05 | 4.5E+05 | 7.5E+05 | 7.0E+05 | 7.1E+05 | 2.1E+05 |
| 7 | 1.50E+06 | 1.70E+06 | 1.75E+06 | 1.85E+06 | 1.70E+06 | 1.5E+05 |
| 14 | 1.75E+06 | 1.70E+06 | 2.25E+06 | 2.50E+06 | 2.05E+06 | 3.9E+05 |
| 20 | 2.90E+06 | 2.25E+06 | 2.60E+06 | 2.35E+06 | 2.53E+06 | 2.9E+05 |
| 27 | 2.75E+06 | 3.00E+06 | 3.25E+06 | 2.85E+06 | 2.96E+06 | 2.2E+05 |
| 34 | 2.50E+06 | 3.35E+06 | 3.20E+06 | 2.90E+06 | 2.99E+06 | 3.8E+05 |
| 41 | 3.05E+06 | 2.80E+06 | 2.20E+06 | 3.00E+06 | 2.76E+06 | 3.9E+05 |
| 51 | 2.1E+06 | 2.8E+06 | 4.2E+06 | 4.6E+06 | 3.4E+06 | 1.2E+06 |
| 54 | 3.25E+06 | 3.15E+06 | 2.90E+06 | 2.05E+06 | 2.84E+06 | 5.5E+05 |
| **Pressure 80 mbar (±2.5mbar)** | |  |  |  |  |  |
| **Incubation days** | **DS1(1)** | **DS1(2)** | **DS2(1)** | **DS2(2)** | **Average** | **Standard Deviation** |
| 0 | 6.8E+04 | 3.7E+04 | 4.0E+04 | 4.4E+04 | 4.7E+04 | 1.4E+04 |
| 7 | 1.378E+05 | 1.433E+05 | 1.422E+05 | 1.411E+05 | 1.411E+05 | 2.4E+03 |
| 14 | 1.40E+05 | 1.80E+05 | 1.10E+05 | 1.50E+05 | 1.45E+05 | 2.9E+04 |
| 25 | 1.80E+05 | 1.20E+05 | 1.60E+05 | 1.50E+05 | 1.53E+05 | 2.5E+04 |
| 32 | 2.00E+05 | 1.80E+05 | 1.50E+05 | 1.90E+05 | 1.80E+05 | 2.2E+04 |
| 42 | 1.10E+05 | 1.70E+05 | 1.70E+05 | 1.60E+05 | 1.53E+05 | 2.9E+04 |
| 49 | 1.80E+05 | 1.10E+05 | 1.50E+05 | 1.40E+05 | 1.45E+05 | 2.9E+04 |
| 56 | 4.0E+04 | 5.0E+04 | 1.60E+05 | 1.40E+05 | 9.7E+04 | 6.1E+04 |

* DS1 and DS2 are the duplicate experiments, and (1) and (2) are the duplicate measurements of each individual culture.

Table S3. Cell counts, mean and Standard deviation measured for duplicate cultures of *Chlorella vulgaris* (CV) measured as cells per ml.

| **Pressure 670 mbar (±20mbar)** | | |  |  |  |  |
| --- | --- | --- | --- | --- | --- | --- |
| **Incubation days** | **CV1(1)** | **CV1(2)** | **CV2(1)** | **CV2(2)** | **Average** | **Standard Deviation** |
| 0 | 1.31E+05 | 1.49E+05 | 1.82E+05 | 1.37E+05 | 1.50E+05 | 2.3E+04 |
| 9 | 1.78E+05 | 1.69E+05 | 1.80E+05 | 2.22E+05 | 1.87E+05 | 2.4E+04 |
| 13 | 2.00E+05 | 1.88E+05 | 2.10E+05 | 1.89E+05 | 1.97E+05 | 1.0E+04 |
| 17 | 2.22E+05 | 2.00E+05 | 1.89E+05 | 2.22E+05 | 2.08E+05 | 1.7E+04 |
| 24 | 2.56E+05 | 2.22E+05 | 2.78E+05 | 2.67E+05 | 2.56E+05 | 2.4E+04 |
| 33 | 2.70E+05 | 2.78E+05 | 2.31E+05 | 2.20E+05 | 2.50E+05 | 2.8E+04 |
| 42 | 3.30E+05 | 2.90E+05 | 3.20E+05 | 3.70E+05 | 3.28E+05 | 3.3E+04 |
| 58 | 3.50E+05 | 3.40E+05 | 3.50E+05 | 3.00E+05 | 3.35E+05 | 2.4E+04 |
| 62 | 3.00E+05 | 3.40E+05 | 3.50E+05 | 3.00E+05 | 3.23E+05 | 2.6E+04 |
| **Pressure 330 mbar (±20mbar)** | | |  |  |  |  |
| **Incubation days** | **CV1(1)** | **CV1(2)** | **CV2(1)** | **CV2(2)** | **Average** | **Standard Deviation** |
| 0 | 1.10E+05 | 1.30E+05 | 1.10E+05 | 1.00E+05 | 1.13E+05 | 1.3E+04 |
| 9 | 5.90E+05 | 6.00E+05 | 7.00E+05 | 5.00E+05 | 5.98E+05 | 8.2E+04 |
| 16 | 7.00E+05 | 7.70E+05 | 8.00E+05 | 7.50E+05 | 7.55E+05 | 4.2E+04 |
| 24 | 7.00E+05 | 8.00E+05 | 8.50E+05 | 7.00E+05 | 7.63E+05 | 7.5E+04 |
| 33 | 8.00E+05 | 8.90E+05 | 8.90E+05 | 8.00E+05 | 8.45E+05 | 5.2E+04 |
| **Pressure 160 mbar (±20mbar)** | | |  |  |  |  |
| **Incubation days** | **CV1(1)** | **CV1(2)** | **CV2(1)** | **CV2(2)** | **Average** | **Standard Deviation** |
| 0 | 1.11E+05 | 1.23E+05 | 1.11E+05 | 1.33E+05 | 1.20E+05 | 1.1E+04 |
| 7 | 1.160E+06 | 1.170E+06 | 1.160E+06 | 1.320E+06 | 1.203E+06 | 7.8E+04 |
| 14 | 1.38E+06 | 1.22E+06 | 1.30E+06 | 1.48E+06 | 1.35E+06 | 1.1E+05 |
| 20 | 1.56E+06 | 1.23E+06 | 1.56E+06 | 1.59E+06 | 1.49E+06 | 1.7E+05 |
| 27 | 1.57E+06 | 1.11E+06 | 1.30E+06 | 1.59E+06 | 1.39E+06 | 2.3E+05 |
| 34 | 1.18E+06 | 1.20E+06 | 1.50E+06 | 1.30E+06 | 1.30E+06 | 1.5E+05 |
| 41 | 1.060E+06 | 1.010E+06 | 8.900E+05 | 1.080E+06 | 1.010E+06 | 8.5E+04 |
| 51 | 9.4E+05 | 6.4E+05 | 1.1E+06 | 9.0E+05 | 8.9E+05 | 1.8E+05 |
| 54 | 9.0E+05 | 9.8E+05 | 6.0E+05 | 1.2E+06 | 9.2E+05 | 2.5E+05 |
| **Pressure 80 mbar (±2.5mbar)** | |  |  |  |  |  |
| **Incubation days** | **CV1(1)** | **CV2(2)** | **CV2(1)** | **CV2(2)** | **Average** | **Standard Deviation** |
| 0 | 8.78E+04 | 1.14E+05 | 9.11E+04 | 1.12E+05 | 1.01E+05 | 1.4E+04 |
| 7 | 1.22E+05 | 1.43E+05 | 1.07E+05 | 1.12E+05 | 1.21E+05 | 1.6E+04 |
| 14 | 3.2E+05 | 5.3E+05 | 4.4E+05 | 5.6E+05 | 4.6E+05 | 1.1E+05 |
| 25 | 4.3E+05 | 4.6E+05 | 7.9E+05 | 6.0E+05 | 5.7E+05 | 1.6E+05 |
| 32 | 5.00E+05 | 5.10E+05 | 7.00E+05 | 6.10E+05 | 5.80E+05 | 9.4E+04 |
| 42 | 5.60E+05 | 5.70E+05 | 4.40E+05 | 6.30E+05 | 5.50E+05 | 8.0E+04 |
| 49 | 5.40E+05 | 5.60E+05 | 5.00E+05 | 5.00E+05 | 5.25E+05 | 3.0E+04 |
| 56 | 6.60E+05 | 6.40E+05 | 6.20E+05 | 6.30E+05 | 6.38E+05 | 1.7E+04 |

* CV1 and CV2 are the duplicate experiments, and (1) and (2) are the duplicate measurements of each individual culture.

**Best fit Logistic growth curve**

Logistic growth of algae cultures growth (cell/ml) was modeled using logistic growth equation and plotted with the average cell counts time series data. The best-fit logistic curve fitting was achieved using the Solver function in Microsoft Excel (v. 16.43). Logistical growth curves were fit by minimizing the residual sum of squares and yielding best-fit T_half_ and slope values, where the mean of cell counts (Y), Standard error (SE) of (Y) Sum of Square of Residuals, Critical T, Degree of freedom and Confidence intervals were generated which are required for Solver function for Best-fit logistic curve fitting (Verschuuren, 2014). Using these parameters, the Solver function solves the correlation matrix to find the best fit values of each parameter by minimizing the residual sum of squares. It is expressed as the units used for the Y- values squared. Standard non-linear logistic fit works by varying the value of model parameters to minimize the value of Residual sum of Squares (Motulsky and Christopoulos, 2004)

Table S4. Best-fit logistical model parameters for *Chloromonas brevispina* (CB) growth used for curve fitting with the Solver function in Microsoft Excel (v. 16.43).

| T_half_ | Slope | Mean of Y | Degree of freedom | Standard Error of Y | Sum of square of Residual | Critical T | Confidence interval |
| --- | --- | --- | --- | --- | --- | --- | --- |
| 14.7 | 0.18 | 8.8E+05 | 5.0 | 1.6E+05 | 1.3000000E+11 | 2.6 | 4.1E+05 |
| 13.9 | 0.18 | 9.2E+05 | 5.0 | 1.6E+05 | 1.2900000E+11 | 2.6 | 4.1E+05 |
| 14.7 | 0.35 | 1.15E+06 | 5.0 | 1.6E+05 | 1.2100000E+11 | 2.6 | 4.0E+05 |
| 14.7 | 0.27 | 1.15E+06 | 5.0 | 1.2E+05 | 7.670000E+10 | 2.6 | 3.2E+05 |
| 17.1 | 0.12 | 5.84E+05 | 7.0 | 6.5E+04 | 2.9900000E+10 | 2.2 | 1.46E+05 |
| 16.7 | 0.13 | 5.66E+05 | 7.0 | 9.0E+04 | 5.6900000E+10 | 2.2 | 2.02E+05 |
| 19.3 | 0.25 | 2.53E+05 | 5.0 | 4.9E+04 | 1.2100000E+10 | 2.6 | 1.26E+05 |
| 20.5 | 0.21 | 2.28E+05 | 5.0 | 3.3E+04 | 5.570000E+09 | 2.6 | 8.6E+04 |

Table S5. Best-fit logistical model parameters for *Dunaliella salina (DS)* growth used for curve fitting with the Solver function in Microsoft Excel (v. 16.43).

| T_half_ | Slope | Mean of Y | Degree of freedom | Standard Error of Y | Sum of square of Residual | Critical T | Confidence interval |
| --- | --- | --- | --- | --- | --- | --- | --- |
| 36.9 | 0.13 | 9.1E+05 | 8.0 | 1.6E+05 | 2.0700000E+11 | 2.3 | 3.7E+05 |
| 38.0 | 0.14 | 8.9E+05 | 8.0 | 2.2E+05 | 3.7500000E+11 | 2.3 | 5.0E+05 |
| 13.0 | 0.50 | 7.15E+05 | 3.0 | 7.9E+04 | 1.8600000E+10 | 3.2 | 2.51E+05 |
| 12.0 | 0.55 | 7.92E+05 | 3.0 | 7.8E+04 | 1.8400000E+10 | 3.2 | 2.49E+05 |
| 7.4 | 0.14 | 2.33E+06 | 7.0 | 2.7E+05 | 5.2600000E+11 | 2.4 | 6.5E+05 |
| 6.5 | 0.14 | 2.54E+06 | 7.0 | 5.8E+05 | 2.32000000E+12 | 2.4 | 1.36E+06 |
| 1.7 | 0.42 | 1.40E+05 | 5.0 | 1.8E+04 | 1.590000E+09 | 2.6 | 4.6E+04 |
| 1.4 | 0.65 | 1.36E+05 | 5.0 | 1.6E+04 | 1.350000E+09 | 2.6 | 4.2E+04 |

Table S6. Best-fit logistical model parameters for *Chlorella vulgaris (CV)* growth used for curve fitting with the Solver function in Microsoft Excel (v. 16.43).

| T_half_ | Slope | Mean of Y | Degree of freedom | Standard Error of Y | Sum of square of Residual | Critical T | Confidence interval |
| --- | --- | --- | --- | --- | --- | --- | --- |
| 6.6 | 0.1 | 2.45E+05 | 7.0 | 1.3E+04 | 1.230000E+09 | 2.4 | 3.1E+04 |
| 3.5 | 0.1 | 2.51E+05 | 7.0 | 3.0E+04 | 6.270000E+09 | 2.4 | 7.1E+04 |
| 6.3 | 0.3 | 6.09E+05 | 3.0 | 6.5E+04 | 1.2600000E+10 | 3.2 | 2.06E+05 |
| 6.2 | 0.3 | 6.20E+05 | 3.0 | 4.2E+04 | 5.230000E+09 | 3.2 | 1.33E+05 |
| 0.5 | 3.4 | 1.08E+06 | 5.0 | 1.4E+05 | 9.220000E+10 | 2.6 | 3.5E+05 |
| 0.6 | 3.2 | 1.17E+06 | 5.0 | 2.1E+05 | 2.1100000E+11 | 2.6 | 5.3E+05 |
| 12.3 | 0.1 | 4.22E+05 | 6.0 | 6.2E+04 | 2.3000000E+10 | 2.5 | 1.51E+05 |
| 9.7 | 0.5 | 4.65E+05 | 6.0 | 9.0E+04 | 4.8300000E+10 | 2.5 | 2.20E+05 |

**Supplementary plots for Logistic growth curve fittings**

**Logistic growth curves fitted with logistic growth equation**

Logistic growth of algae cultures growth (cell/ml) was modeled using logistic growth equation and plotted with the average cell counts time series data. The best-fit logistic curve fitting was achieved using the Solver function in Microsoft Excel (v. 16.43).

Figure S1. Best-fit logistic curve fitting for *Chloromonas brevispina (CB1)* cell/ml at 670 mbar

Figure S2. Best-fit logistic curve fitting for *Chloromonas brevispina (CB2)* cell/ml at 670 mbar

Figure S3. Best-fit logistic curve fitting for *Chloromonas brevispina (CB1)* cell/ml at 330 mbar

Figure S4. Best-fit logistic curve fitting for *Chloromonas brevispina (CB2)* cell/ml at 330 mbar

| 670mbar | CB1 |  |  |  |
| --- | --- | --- | --- | --- |
| Days | CB1 (avg) | Logistic growth | Upper CI | Lower CI |
| 0 | 1.06E+05 | 1.13E+05 | 5.28E+05 | -3.01E+05 |
| 3 | 2.68E+05 | 1.84E+05 | 5.98E+05 | -2.31E+05 |
| 10 | 5.75E+05 | 4.93E+05 | 9.08E+05 | 7.83E+04 |
| 17 | 7.25E+05 | 9.71E+05 | 1.39E+06 | 5.56E+05 |
| 19 | 1.25E+06 | 1.10E+06 | 1.52E+06 | 6.86E+05 |
| 26 | 1.58E+06 | 1.42E+06 | 1.83E+06 | 1.01E+06 |
| 33 | 1.65E+06 | 1.55E+06 | 1.97E+06 | 1.14E+06 |

Figure S5. Best-fit logistic curve fitting for *Chloromonas brevispina (CB1)* cell/ml at 160 mbar

| 670mbar | CB1 |  |  |  |
| --- | --- | --- | --- | --- |
| Days | CB1 (avg) | Logistic growth | Upper CI | Lower CI |
| 0 | 1.06E+05 | 1.13E+05 | 5.28E+05 | -3.01E+05 |
| 3 | 2.68E+05 | 1.84E+05 | 5.98E+05 | -2.31E+05 |
| 10 | 5.75E+05 | 4.93E+05 | 9.08E+05 | 7.83E+04 |
| 17 | 7.25E+05 | 9.71E+05 | 1.39E+06 | 5.56E+05 |
| 19 | 1.25E+06 | 1.10E+06 | 1.52E+06 | 6.86E+05 |
| 26 | 1.58E+06 | 1.42E+06 | 1.83E+06 | 1.01E+06 |
| 33 | 1.65E+06 | 1.55E+06 | 1.97E+06 | 1.14E+06 |

Figure S6. Best-fit logistic curve fitting for *Chloromonas brevispina (CB2)* cell/ml at 160 mbar

| 670mbar | CB1 |  |  |  |
| --- | --- | --- | --- | --- |
| Days | CB1 (avg) | Logistic growth | Upper CI | Lower CI |
| 0 | 1.06E+05 | 1.13E+05 | 5.28E+05 | -3.01E+05 |
| 3 | 2.68E+05 | 1.84E+05 | 5.98E+05 | -2.31E+05 |
| 10 | 5.75E+05 | 4.93E+05 | 9.08E+05 | 7.83E+04 |
| 17 | 7.25E+05 | 9.71E+05 | 1.39E+06 | 5.56E+05 |
| 19 | 1.25E+06 | 1.10E+06 | 1.52E+06 | 6.86E+05 |
| 26 | 1.58E+06 | 1.42E+06 | 1.83E+06 | 1.01E+06 |
| 33 | 1.65E+06 | 1.55E+06 | 1.97E+06 | 1.14E+06 |

Figure S7. Best-fit logistic curve fitting for *Chloromonas brevispina (CB2)* cell/ml at 80 mbar

Figure S8. Best-fit logistic curve fitting for *Chloromonas brevispina (CB2)* cell/ml at 80 mbar

| 670mbar | CB1 |  |  |  |
| --- | --- | --- | --- | --- |
| Days | CB1 (avg) | Logistic growth | Upper CI | Lower CI |
| 0 | 1.06E+05 | 1.13E+05 | 5.28E+05 | -3.01E+05 |
| 3 | 2.68E+05 | 1.84E+05 | 5.98E+05 | -2.31E+05 |
| 10 | 5.75E+05 | 4.93E+05 | 9.08E+05 | 7.83E+04 |
| 17 | 7.25E+05 | 9.71E+05 | 1.39E+06 | 5.56E+05 |
| 19 | 1.25E+06 | 1.10E+06 | 1.52E+06 | 6.86E+05 |
| 26 | 1.58E+06 | 1.42E+06 | 1.83E+06 | 1.01E+06 |
| 33 | 1.65E+06 | 1.55E+06 | 1.97E+06 | 1.14E+06 |

**Logistic growth curve of *Dunaliella salina* (DS)**

Figure S9. Best-fit logistic curve fitting for *Dunaliella salina (DS1)* cell/ml at 670 mbar

| 670mbar | CB1 |  |  |  |
| --- | --- | --- | --- | --- |
| Days | CB1 (avg) | Logistic growth | Upper CI | Lower CI |
| 0 | 1.06E+05 | 1.13E+05 | 5.28E+05 | -3.01E+05 |
| 3 | 2.68E+05 | 1.84E+05 | 5.98E+05 | -2.31E+05 |
| 10 | 5.75E+05 | 4.93E+05 | 9.08E+05 | 7.83E+04 |
| 17 | 7.25E+05 | 9.71E+05 | 1.39E+06 | 5.56E+05 |
| 19 | 1.25E+06 | 1.10E+06 | 1.52E+06 | 6.86E+05 |
| 26 | 1.58E+06 | 1.42E+06 | 1.83E+06 | 1.01E+06 |
| 33 | 1.65E+06 | 1.55E+06 | 1.97E+06 | 1.14E+06 |

Figure S10. Best-fit logistic curve fitting for *Dunaliella salina (DS2)* cell/ml at 670 mbar

| 670mbar | CB1 |  |  |  |
| --- | --- | --- | --- | --- |
| Days | CB1 (avg) | Logistic growth | Upper CI | Lower CI |
| 0 | 1.06E+05 | 1.13E+05 | 5.28E+05 | -3.01E+05 |
| 3 | 2.68E+05 | 1.84E+05 | 5.98E+05 | -2.31E+05 |
| 10 | 5.75E+05 | 4.93E+05 | 9.08E+05 | 7.83E+04 |
| 17 | 7.25E+05 | 9.71E+05 | 1.39E+06 | 5.56E+05 |
| 19 | 1.25E+06 | 1.10E+06 | 1.52E+06 | 6.86E+05 |
| 26 | 1.58E+06 | 1.42E+06 | 1.83E+06 | 1.01E+06 |
| 33 | 1.65E+06 | 1.55E+06 | 1.97E+06 | 1.14E+06 |

Figure S11. Best-fit logistic curve fitting for *Dunaliella salina (DS1)* cell/ml at 330 mbar

| 670mbar | CB1 |  |  |  |
| --- | --- | --- | --- | --- |
| Days | CB1 (avg) | Logistic growth | Upper CI | Lower CI |
| 0 | 1.06E+05 | 1.13E+05 | 5.28E+05 | -3.01E+05 |
| 3 | 2.68E+05 | 1.84E+05 | 5.98E+05 | -2.31E+05 |
| 10 | 5.75E+05 | 4.93E+05 | 9.08E+05 | 7.83E+04 |
| 17 | 7.25E+05 | 9.71E+05 | 1.39E+06 | 5.56E+05 |
| 19 | 1.25E+06 | 1.10E+06 | 1.52E+06 | 6.86E+05 |
| 26 | 1.58E+06 | 1.42E+06 | 1.83E+06 | 1.01E+06 |
| 33 | 1.65E+06 | 1.55E+06 | 1.97E+06 | 1.14E+06 |

Figure S12. Best-fit logistic curve fitting for *Dunaliella salina (DS2)* cell/ml at 330 mbar

| 670mbar | CB1 |  |  |  |
| --- | --- | --- | --- | --- |
| Days | CB1 (avg) | Logistic growth | Upper CI | Lower CI |
| 0 | 1.06E+05 | 1.13E+05 | 5.28E+05 | -3.01E+05 |
| 3 | 2.68E+05 | 1.84E+05 | 5.98E+05 | -2.31E+05 |
| 10 | 5.75E+05 | 4.93E+05 | 9.08E+05 | 7.83E+04 |
| 17 | 7.25E+05 | 9.71E+05 | 1.39E+06 | 5.56E+05 |
| 19 | 1.25E+06 | 1.10E+06 | 1.52E+06 | 6.86E+05 |
| 26 | 1.58E+06 | 1.42E+06 | 1.83E+06 | 1.01E+06 |
| 33 | 1.65E+06 | 1.55E+06 | 1.97E+06 | 1.14E+06 |

Figure S13. Best-fit logistic curve fitting for *Dunaliella salina (DS1)* cell/ml at 160 mbar

| 670mbar | CB1 |  |  |  |
| --- | --- | --- | --- | --- |
| Days | CB1 (avg) | Logistic growth | Upper CI | Lower CI |
| 0 | 1.06E+05 | 1.13E+05 | 5.28E+05 | -3.01E+05 |
| 3 | 2.68E+05 | 1.84E+05 | 5.98E+05 | -2.31E+05 |
| 10 | 5.75E+05 | 4.93E+05 | 9.08E+05 | 7.83E+04 |
| 17 | 7.25E+05 | 9.71E+05 | 1.39E+06 | 5.56E+05 |
| 19 | 1.25E+06 | 1.10E+06 | 1.52E+06 | 6.86E+05 |
| 26 | 1.58E+06 | 1.42E+06 | 1.83E+06 | 1.01E+06 |
| 33 | 1.65E+06 | 1.55E+06 | 1.97E+06 | 1.14E+06 |

Figure S14. Best-fit logistic curve fitting for *Dunaliella salina (DS2)* cell/ml at 160 mbar

| 670mbar | CB1 |  |  |  |
| --- | --- | --- | --- | --- |
| Days | CB1 (avg) | Logistic growth | Upper CI | Lower CI |
| 0 | 1.06E+05 | 1.13E+05 | 5.28E+05 | -3.01E+05 |
| 3 | 2.68E+05 | 1.84E+05 | 5.98E+05 | -2.31E+05 |
| 10 | 5.75E+05 | 4.93E+05 | 9.08E+05 | 7.83E+04 |
| 17 | 7.25E+05 | 9.71E+05 | 1.39E+06 | 5.56E+05 |
| 19 | 1.25E+06 | 1.10E+06 | 1.52E+06 | 6.86E+05 |
| 26 | 1.58E+06 | 1.42E+06 | 1.83E+06 | 1.01E+06 |
| 33 | 1.65E+06 | 1.55E+06 | 1.97E+06 | 1.14E+06 |

Figure S15. Best-fit logistic curve fitting for *Dunaliella salina (DS1)* cell/ml at 80 mbar

| 670mbar | CB1 |  |  |  |
| --- | --- | --- | --- | --- |
| Days | CB1 (avg) | Logistic growth | Upper CI | Lower CI |
| 0 | 1.06E+05 | 1.13E+05 | 5.28E+05 | -3.01E+05 |
| 3 | 2.68E+05 | 1.84E+05 | 5.98E+05 | -2.31E+05 |
| 10 | 5.75E+05 | 4.93E+05 | 9.08E+05 | 7.83E+04 |
| 17 | 7.25E+05 | 9.71E+05 | 1.39E+06 | 5.56E+05 |
| 19 | 1.25E+06 | 1.10E+06 | 1.52E+06 | 6.86E+05 |
| 26 | 1.58E+06 | 1.42E+06 | 1.83E+06 | 1.01E+06 |
| 33 | 1.65E+06 | 1.55E+06 | 1.97E+06 | 1.14E+06 |

Figure S16. Best-fit logistic curve fitting for *Dunaliella salina (DS2)* cell/ml at 80 mbar

| 670mbar | CB1 |  |  |  |
| --- | --- | --- | --- | --- |
| Days | CB1 (avg) | Logistic growth | Upper CI | Lower CI |
| 0 | 1.06E+05 | 1.13E+05 | 5.28E+05 | -3.01E+05 |
| 3 | 2.68E+05 | 1.84E+05 | 5.98E+05 | -2.31E+05 |
| 10 | 5.75E+05 | 4.93E+05 | 9.08E+05 | 7.83E+04 |
| 17 | 7.25E+05 | 9.71E+05 | 1.39E+06 | 5.56E+05 |
| 19 | 1.25E+06 | 1.10E+06 | 1.52E+06 | 6.86E+05 |
| 26 | 1.58E+06 | 1.42E+06 | 1.83E+06 | 1.01E+06 |
| 33 | 1.65E+06 | 1.55E+06 | 1.97E+06 | 1.14E+06 |

**Logistic growth curve of *Chlorella vulgaris (*CV)**

Figure S17. Best-fit logistic curve fitting for *Chlorella vulgaris (CV1)* cell/ml at 670 mbar

| 670mbar | CB1 |  |  |  |
| --- | --- | --- | --- | --- |
| Days | CB1 (avg) | Logistic growth | Upper CI | Lower CI |
| 0 | 1.06E+05 | 1.13E+05 | 5.28E+05 | -3.01E+05 |
| 3 | 2.68E+05 | 1.84E+05 | 5.98E+05 | -2.31E+05 |
| 10 | 5.75E+05 | 4.93E+05 | 9.08E+05 | 7.83E+04 |
| 17 | 7.25E+05 | 9.71E+05 | 1.39E+06 | 5.56E+05 |
| 19 | 1.25E+06 | 1.10E+06 | 1.52E+06 | 6.86E+05 |
| 26 | 1.58E+06 | 1.42E+06 | 1.83E+06 | 1.01E+06 |
| 33 | 1.65E+06 | 1.55E+06 | 1.97E+06 | 1.14E+06 |

| 670mbar | CB1 |  |  |  |
| --- | --- | --- | --- | --- |
| Days | CB1 (avg) | Logistic growth | Upper CI | Lower CI |
| 0 | 1.06E+05 | 1.13E+05 | 5.28E+05 | -3.01E+05 |
| 3 | 2.68E+05 | 1.84E+05 | 5.98E+05 | -2.31E+05 |
| 10 | 5.75E+05 | 4.93E+05 | 9.08E+05 | 7.83E+04 |
| 17 | 7.25E+05 | 9.71E+05 | 1.39E+06 | 5.56E+05 |
| 19 | 1.25E+06 | 1.10E+06 | 1.52E+06 | 6.86E+05 |
| 26 | 1.58E+06 | 1.42E+06 | 1.83E+06 | 1.01E+06 |
| 33 | 1.65E+06 | 1.55E+06 | 1.97E+06 | 1.14E+06 |

Figure S18. Best-fit logistic curve fitting for *Chlorella vulgaris (CV2)* cell/ml at 670 mbar

Figure S19. Best-fit logistic curve fitting for *Chlorella vulgaris (CV1)* cell/ml at 330 mbar

Figure S20. Best-fit logistic curve fitting for *Chlorella vulgaris (CV2)* cell/ml at 330 mbar

| 670mbar | CB1 |  |  |  |
| --- | --- | --- | --- | --- |
| Days | CB1 (avg) | Logistic growth | Upper CI | Lower CI |
| 0 | 1.06E+05 | 1.13E+05 | 5.28E+05 | -3.01E+05 |
| 3 | 2.68E+05 | 1.84E+05 | 5.98E+05 | -2.31E+05 |
| 10 | 5.75E+05 | 4.93E+05 | 9.08E+05 | 7.83E+04 |
| 17 | 7.25E+05 | 9.71E+05 | 1.39E+06 | 5.56E+05 |
| 19 | 1.25E+06 | 1.10E+06 | 1.52E+06 | 6.86E+05 |
| 26 | 1.58E+06 | 1.42E+06 | 1.83E+06 | 1.01E+06 |
| 33 | 1.65E+06 | 1.55E+06 | 1.97E+06 | 1.14E+06 |

Figure S21. Best-fit logistic curve fitting for *Chlorella vulgaris (CV1)* cell/ml at 160 mbar

| 670mbar | CB1 |  |  |  |
| --- | --- | --- | --- | --- |
| Days | CB1 (avg) | Logistic growth | Upper CI | Lower CI |
| 0 | 1.06E+05 | 1.13E+05 | 5.28E+05 | -3.01E+05 |
| 3 | 2.68E+05 | 1.84E+05 | 5.98E+05 | -2.31E+05 |
| 10 | 5.75E+05 | 4.93E+05 | 9.08E+05 | 7.83E+04 |
| 17 | 7.25E+05 | 9.71E+05 | 1.39E+06 | 5.56E+05 |
| 19 | 1.25E+06 | 1.10E+06 | 1.52E+06 | 6.86E+05 |
| 26 | 1.58E+06 | 1.42E+06 | 1.83E+06 | 1.01E+06 |
| 33 | 1.65E+06 | 1.55E+06 | 1.97E+06 | 1.14E+06 |

| Days | CV2 (avg) | Logistic growth | Upper CI | Lower CI |
| --- | --- | --- | --- | --- |
| 0 | 1.22E+05 | 1.67E+05 | 6.95E+05 | -3.62E+05 |
| 7 | 1.24E+06 | 1.35E+06 | 1.88E+06 | 8.23E+05 |
| 14 | 1.39E+06 | 1.35E+06 | 1.88E+06 | 8.23E+05 |
| 20 | 1.58E+06 | 1.35E+06 | 1.88E+06 | 8.23E+05 |
| 27 | 1.45E+06 | 1.35E+06 | 1.88E+06 | 8.23E+05 |
| 34 | 1.40E+06 | 1.35E+06 | 1.88E+06 | 8.23E+05 |
| 41 | 9.85E+05 | 1.35E+06 | 1.88E+06 | 8.23E+05 |

Figure S22. Best-fit logistic curve fitting for *Chlorella vulgaris (CV2)* cell/ml at 160 mbar

| 670mbar | CB1 |  |  |  |
| --- | --- | --- | --- | --- |
| Days | CB1 (avg) | Logistic growth | Upper CI | Lower CI |
| 0 | 1.06E+05 | 1.13E+05 | 5.28E+05 | -3.01E+05 |
| 3 | 2.68E+05 | 1.84E+05 | 5.98E+05 | -2.31E+05 |
| 10 | 5.75E+05 | 4.93E+05 | 9.08E+05 | 7.83E+04 |
| 17 | 7.25E+05 | 9.71E+05 | 1.39E+06 | 5.56E+05 |
| 19 | 1.25E+06 | 1.10E+06 | 1.52E+06 | 6.86E+05 |
| 26 | 1.58E+06 | 1.42E+06 | 1.83E+06 | 1.01E+06 |
| 33 | 1.65E+06 | 1.55E+06 | 1.97E+06 | 1.14E+06 |

Figure S23. Best-fit logistic curve fitting for *Chlorella vulgaris (CV1)* cell/ml at 80 mbar

Figure S24. Best-fit logistic curve fitting for *Chlorella vulgaris (CV2)* cell/ml at 80 mbar

| 670mbar | CB1 |  |  |  |
| --- | --- | --- | --- | --- |
| Days | CB1 (avg) | Logistic growth | Upper CI | Lower CI |
| 0 | 1.06E+05 | 1.13E+05 | 5.28E+05 | -3.01E+05 |
| 3 | 2.68E+05 | 1.84E+05 | 5.98E+05 | -2.31E+05 |
| 10 | 5.75E+05 | 4.93E+05 | 9.08E+05 | 7.83E+04 |
| 17 | 7.25E+05 | 9.71E+05 | 1.39E+06 | 5.56E+05 |
| 19 | 1.25E+06 | 1.10E+06 | 1.52E+06 | 6.86E+05 |
| 26 | 1.58E+06 | 1.42E+06 | 1.83E+06 | 1.01E+06 |
| 33 | 1.65E+06 | 1.55E+06 | 1.97E+06 | 1.14E+06 |

**Supplementary tables for Optical Density measurements**

Table S7. Optical density (OD) and Mean (Avg) measured for duplicate cultures of *Chloromonas brevispina* *(CB)* and *Kremastochrysopsis austriaca (KC).*

| **Pressure 670 mbar (±20mbar)** | | | | | | |
| --- | --- | --- | --- | --- | --- | --- |
| **Time (days)** | **CB1**  **(OD)** | **CB2**  **(OD)** | **Avg**  **(OD)** | **KC1**  **(OD)** | **KC2**  **(OD)** | **Avg**  **(OD)** |
| 0 | 0.04 | 0.04 | 0.04 | 0.02 | 0.02 | 0.02 |
| 3 | 0.05 | 0.05 | 0.05 | 0.02 | 0.02 | 0.02 |
| 10 | 0.06 | 0.07 | 0.07 | 0.02 | 0.02 | 0.02 |
| 17 | 0.30 | 0.34 | 0.32 | 0.03 | 0.03 | 0.03 |
| 19 | 0.32 | 0.41 | 0.37 | 0.03 | 0.03 | 0.03 |
| 26 | 0.32 | 0.41 | 0.36 | 0.04 | 0.04 | 0.04 |
| 33 | 0.42 | 0.45 | 0.44 | 0.04 | 0.04 | 0.04 |
| **Pressure 330 mbar (±20mbar)** | | | | | | |
| **Time (days)** | **CB1**  **(OD)** | **CB2**  **(OD)** | **Avg**  **(OD)** | **KC1**  **(OD)** | **KC2**  **(OD)** | **Avg**  **(OD)** |
| 0 | 0.05 | 0.05 | 0.05 | 0.02 | 0.02 | 0.02 |
| 7 | 0.08 | 0.10 | 0.09 | 0.04 | 0.04 | 0.04 |
| 14 | 0.23 | 0.20 | 0.22 | 0.03 | 0.04 | 0.04 |
| 20 | 0.40 | 0.31 | 0.35 | 0.04 | 0.04 | 0.04 |
| 23 | 0.41 | 0.31 | 0.36 | 0.03 | 0.04 | 0.03 |
| 30 | 0.44 | 0.40 | 0.42 | 0.04 | 0.04 | 0.04 |
| 36 | 0.46 | 0.42 | 0.44 | 0.04 | 0.04 | 0.04 |
| **Pressure 160 mbar (±20mbar)** | | | | | | |
| **Time (days)** | **CB1**  **(OD)** | **CB2**  **(OD)** | **Avg**  **(OD)** | **KC1**  **(OD)** | **KC2**  **(OD)** | **Avg**  **(OD)** |
| 0 | 0.02 | 0.02 | 0.02 | 0.01 | 0.02 | 0.01 |
| 7 | 0.03 | 0.04 | 0.03 | 0.02 | 0.03 | 0.02 |
| 14 | 0.10 | 0.10 | 0.10 | 0.03 | 0.03 | 0.03 |
| 20 | 0.10 | 0.09 | 0.10 | 0.03 | 0.04 | 0.04 |
| 27 | 0.10 | 0.10 | 0.10 | 0.03 | 0.03 | 0.03 |
| 34 | 0.19 | 0.20 | 0.19 | 0.02 | 0.04 | 0.03 |
| 41 | 0.21 | 0.20 | 0.21 | 0.02 | 0.02 | 0.02 |
| 51 | 0.20 | 0.19 | 0.20 | 0.05 | 0.09 | 0.07 |
| 54 | 0.24 | 0.23 | 0.23 | 0.06 | 0.10 | 0.08 |
| **Pressure 80 mbar (±2.5mbar)** | | | | | | |
| **Time (days)** | **CB1**  **(OD)** | **CB2**  **(OD)** | **Avg**  **(OD)** | **KC1**  **(OD)** | **KC2**  **(OD)** | **Avg**  **(OD)** |
| 0 | 0.01 | 0.01 | 0.01 | 0.01 | 0.01 | 0.01 |
| 7 | 0.03 | 0.01 | 0.02 | 0.01 | 0.01 | 0.01 |
| 14 | 0.02 | 0.02 | 0.02 | 0.00 | 0.00 | 0.00 |
| 21 | 0.11 | 0.10 | 0.11 | 0.01 | 0.01 | 0.01 |
| 28 | 0.13 | 0.13 | 0.13 | 0.00 | 0.01 | 0.01 |
| 35 | 0.15 | 0.16 | 0.16 | 0.01 | 0.01 | 0.01 |
| 42 | 0.16 | 0.16 | 0.16 | 0.01 | 0.01 | 0.01 |

Table S8. Optical density (OD) and Mean (Avg) measured for duplicate cultures of *Chlorella vulgaris* (*CV*), *Dunaliella salina (DS)* and *Spirulina plantensis (SP).*

| **Pressure 670 mbar (±20mbar)** | | | | | | | | | |
| --- | --- | --- | --- | --- | --- | --- | --- | --- | --- |
| **Time (days)** | **DS1** | **DS2** | **Avg**  **(OD)** | **CV1** | **CV2** | **Avg**  **(OD)** | **SP1** | **SP2** | **Avg**  **(OD)** |
| 0 | 0.01 | 0.01 | 0.01 | 0.02 | 0.02 | 0.02 | 0.01 | 0.01 | 0.01 |
| 9 | 0.03 | 0.03 | 0.03 | 0.14 | 0.10 | 0.12 | 0.02 | 0.01 | 0.02 |
| 13 | 0.04 | 0.03 | 0.04 | 0.18 | 0.19 | 0.19 | 0.01 | 0.01 | 0.01 |
| 17 | 0.04 | 0.04 | 0.04 | 0.28 | 0.19 | 0.24 | 0.01 | 0.01 | 0.01 |
| 24 | 0.05 | 0.04 | 0.04 | 0.33 | 0.31 | 0.32 | 0.01 | 0.01 | 0.01 |
| 33 | 0.35 | 0.38 | 0.37 | 0.31 | 0.30 | 0.31 | 0.00 | 0.01 | 0.00 |
| 42 | 0.35 | 0.40 | 0.38 | 0.31 | 0.32 | 0.32 | 0.01 | 0.01 | 0.01 |
| 49 | 0.65 | 0.67 | 0.66 | 0.30 | 0.30 | 0.30 | 0.01 | 0.01 | 0.01 |
| 58 | 0.70 | 0.65 | 0.68 | 0.35 | 0.30 | 0.33 | 0.01 | 0.01 | 0.01 |
| 62 | 0.69 | 0.68 | 0.68 | 0.30 | 0.45 | 0.38 | 0.01 | 0.01 | 0.01 |
| **Pressure 330 mbar (±20mbar)** | | | | | | | | | |
| **Time (days)** | **DS1** | **DS2** | **Avg**  **(OD)** | **CV1** | **CV2** | **Avg**  **(OD)** | **SP1** | **SP2** | **Avg**  **(OD)** |
| 0 | 0.04 | 0.02 | 0.03 | 0.02 | 0.01 | 0.01 | 0.00 | 0.00 | 0.00 |
| 9 | 0.03 | 0.03 | 0.03 | 0.20 | 0.20 | 0.20 | 0.00 | 0.00 | 0.00 |
| 16 | 0.29 | 0.30 | 0.30 | 0.20 | 0.21 | 0.21 | 0.00 | 0.00 | 0.00 |
| 24 | 0.34 | 0.33 | 0.34 | 0.23 | 0.25 | 0.24 | 0.00 | 0.00 | 0.00 |
| 33 | 0.35 | 0.35 | 0.35 | 0.33 | 0.32 | 0.33 | 0.00 | 0.00 | 0.00 |
| **Pressure 160 mbar (±20mbar)** | | | | | | |  |  |  |
| **Time (days)** | **DS1** | **DS2** | **Avg**  **(OD)** | **CV1** | **CV2** | **Avg**  **(OD)** |  |  |  |
| 0 | 0.05 | 0.05 | 0.05 | 0.09 | 0.10 | 0.10 |  |  |  |
| 7 | 0.30 | 0.32 | 0.31 | 0.20 | 0.28 | 0.24 |  |  |  |
| 14 | 0.83 | 0.84 | 0.83 | 0.24 | 0.28 | 0.26 |  |  |  |
| 20 | 1.07 | 0.97 | 1.02 | 0.23 | 0.24 | 0.24 |  |  |  |
| 27 | 1.00 | 0.98 | 0.99 | 0.20 | 0.24 | 0.22 |  |  |  |
| 34 | 0.99 | 0.93 | 0.96 | 0.18 | 0.18 | 0.18 |  |  |  |
| 41 | 1.50 | 1.10 | 1.30 | 0.09 | 0.12 | 0.11 |  |  |  |
| 51 | 0.55 | 0.60 | 0.58 | 0.11 | 0.09 | 0.10 |  |  |  |
| 54 | 0.58 | 0.61 | 0.60 | 0.10 | 0.10 | 0.10 |  |  |  |
| **Pressure 80 mbar (±2.5mbar)** | | | | | | |  |  |  |
| **Time (day)** | **DS1** | **DS2** | **Avg**  **(OD)** | **CV1** | **CV2** | **Avg**  **(OD)** |  |  |  |
| 0 | 0.03 | 0.02 | 0.03 | 0.02 | 0.02 | 0.02 |  |  |  |
| 7 | 0.07 | 0.06 | 0.07 | 0.04 | 0.04 | 0.04 |  |  |  |
| 14 | 0.07 | 0.07 | 0.07 | 0.05 | 0.06 | 0.06 |  |  |  |
| 25 | 0.09 | 0.09 | 0.09 | 0.07 | 0.07 | 0.07 |  |  |  |
| 32 | 0.15 | 0.12 | 0.14 | 0.08 | 0.09 | 0.09 |  |  |  |
| 42 | 0.20 | 0.19 | 0.20 | 0.15 | 0.16 | 0.16 |  |  |  |
| 49 | 0.11 | 0.15 | 0.13 | 0.15 | 0.14 | 0.15 |  |  |  |
| 56 | 0.08 | 0.06 | 0.07 | 0.13 | 0.13 | 0.13 |  |  |  |

**Supplementary Data for statistical analysis**

Table S9: P-value and R^2^ calculated from the growth of C*hloromonas brevispina (CB), Kremastochrysopsis austriaca, (KC), Dunaliella salina (DS), Chlorella vulgaris (CV),* and *Spirulina plantensis (SP*), measured at 670 mbar plotted as average of the duplicates values of OD measurements against the time (days) using Microsoft Excel Analysis Toolkit.

| Algae species | P-value | R^2^ |
| --- | --- | --- |
| CB | 3.1E-03 | 0.93 |
| KC | 2.4E-04 | 0.94 |
| DS | 1.2E-05 | 0.90 |
| CV | 1.9E-02 | 0.88 |
| SP | 2.0E-01 | 0.33 |

Figure S25: Best-fit exponential models fit to average of the duplicate values of OD measurements of algae growing at 670 mbar data spanning from day 0 to one data point past T_half_.

a)

**T_half_ =13.59**

b)

c)

d)

e)

Table S10: R^2^ calculated from the Best-fit exponential growth model of C*hloromonas brevispina (CB), Dunaliella salina (DS) and Chlorella vulgaris (CV)* cultures measured at different pressures plotted as log scale values of average of the two cell counts/ ml measurements of same culture against the time (days) using Microsoft Excel Analysis Toolkit.

| Pressure (mbar) | C*hloromonas brevispina (CB)* | | *Dunaliella salina*  *(DS)* | | *Chlorella vulgaris*  *(CV)* | |
| --- | --- | --- | --- | --- | --- | --- |
|  | R^2^ | Observations  (n) | R^2^ | Observations  (n) | R^2^ | Observations  (n) |
| 670 ±20 (1) | 0.86 | 4 | 0.73 | 6 | 1.00 | 3 |
| 670 ±20 (2) | 0.91 | 4 | 0.87 | 6 | 0.89 | 3 |
| 330 ±20 (1) | 0.89 | 4 | 0.92 | 3 | 0.89 | 3 |
| 330 ±20 (2) | 0.86 | 4 | 0.99 | 3 | 0.89 | 3 |
| 160 ±20(1) | 0.93 | 4 | 0.81 | 3 | 0.78 | 3 |
| 160 ±20 (2) | 0.86 | 4 | 0.91 | 3 | 0.79 | 3 |
| 80 ±2.5 (1) | 0.94 | 4 | 0.84 | 3 | 0.89 | 3 |
| 80 ±2.5 (2) | 0.83 | 4 | 0.69 | 3 | 0.78 | 3 |

Figure S26: A) Modified low pressure chamber, held 670 mbar pressure for 36 hours, after which, it was pumped down to the required pressure. It could not hold it at 330 mbar or lower pressures. B) Aluminum Vacuum Chamber (Slickvacseal) used for algae growth experiments at 330 mbar, 160 mbar and 80 mbar and was able to hold the low pressures for a week.

Table S11. Table showing the carrying capacities (*C_algae_, _max_)* computed at different pressures as the average of two cell counts of algae *Chlorella vulgaris* (CV) *Dunaliella salina (DS)* and *Chloromonas brevispina* (*CB*).

| Pressure (mbar) | Average carrying capacity of duplicates (*C_algae_, _max_)* | Uncertainty | n (number of time points the average carrying capacity is based on) |
| --- | --- | --- | --- |
| ***Chloromonas brevispina*** | | | |
| 670 ±20 (1) | 1.613E+06 | 5.3E+04 | 2 |
| 670 ±20 (2) | 1.613E+06 | 8.8E+04 | 2 |
| 330 ±20 (1) | 2.11E+06 | 1.4E+05 | 3 |
| 330 ±20 (2) | 1.85E+06 | 1.1E+05 | 3 |
| 160 ±20 (1) | 8.95E+05 | 5.1E+04 | 3 |
| 160 ±20 (2) | 8.4E+05 | 1.1E+05 | 3 |
| 80 ±2.5 (1) | 4.52E+05 | 4.6E+04 | 3 |
| 80 ±2.5 (2) | 4.17E+05 | 2.1E+04 | 3 |
| ***Dunaliella salina*** | | | |
| 670 ±20 (1) | 5.3E+04 | N/A^*^ | 1 |
| 670 ±20 (2) | 8.8E+04 | N/A^*^ | 1 |
| 330 ±20 (1) | 1.4E+05 | 1.1E+05 | 3 |
| 330 ±20 (2) | 1.1E+05 | 1.1E+05 | 3 |
| 160 ±20 (1) | 5.07E+04 | 3.2E+05 | 4 |
| 160 ±20 (2) | 1.1E+05 | 8.6E+05 | 4 |
| 80 ±2.5 (1) | 4.6E+04 | 2.3E+04 | 4 |
| 80 ±2.5 (2) | 2.1E+04 | 1.1E+04 | 4 |
| ***Chlorella vulgaris*** | | | |
| 670 ±20 (1) | 3.25E+05 | 1.8E+04 | 3 |
| 670 ±20 (2) | 3.32E+05 | 1.2E+04 | 3 |
| 330 ±20 (1) | 7.77E+05 | 6.0E+04 | 3 |
| 330 ±20 (2) | 7.98E+05 | 4.0E+04 | 3 |
| 160 ±20 (1) | 1.24E+06 | 1.6E+05 | 4 |
| 160 ±20 (2) | 1.35E+06 | 2.6E+05 | 4 |
| 80 ±2.5 (1) | 5.88E+05 | 5.4E+04 | 3 |
| 80 ±2.5 (2) | 5.53E+05 | 7.3E+04 | 3 |
|  |  |  |  |

The carrying capacity values for *Chloromonas brevispina (CB),* *D salina (DS)* and *Chlorella vulgaris (CV)* were calculated from the average of 2-4 time points except for *D. salina* for which 1 point was used for the average of two cell counts for each experiment. The uncertainties are the 1 standard deviation of the mean where 3-4 time points were used, and the range between the values where two points were used.

Table S12. Best-fit exponential model parameters computed for the duplicate cultures of candidate algae at different pressures.

| **Pressure**  **(mbar)** | ***Chloromonas brevispina***  ***(CB)*** | | | | ***Dunaliella salina***  ***(DS)*** | | | | ***Chlorella vulgaris***  ***(CV)*** | | | |
| --- | --- | --- | --- | --- | --- | --- | --- | --- | --- | --- | --- | --- |
|  | **^a^ LPD** | **^b^ r** | **^c^ T_d_** | **^d^ R^2^** | **^a^LPD** | **^b^ r** | **^c^ T_d_** | **^d^ R^2^** | **^a^ LPD** | **^b^ r** | **^c^ T_d_** | **^d^ R^2^** |
| **670±20 (1)** | 7.0 ±0.3 | 0.11±0.03 | 6.5 ±2.1 | 0.86 | 25±2.0 | 0.09 ±0.02 | 8.7±3.0 | 0.73 | 8.5 ±0.1 | 0.02±0.00 | 28 ±1.1 | 1 |
| **670±20 (2)** | 6.7 ±0.3 | 0.11 ±0.03 | 6.3 ±1.5 | 0.91 | 27±2.0 | 0.08 ±0.01 | 8.5±1.5 | 0.87 | 8.7 ±0.1 | 0.02±0.01 | 37 ±14 | 0.89 |
| **330±20 (1)** | 11.2±1.0 | 0.11 ±0.03 | 6.3 ±1.5 | 0.91 | 10±1.0 | 0.08 ±0.01 | 4.0±1.3 | 0.87 | 2.5 ±0.2 | 0.12±0.04 | 6.0 ±2.5 | 0.89 |
| **330 ±20(2)** | 10.2 ±1.0 | 0.12 ±0.03 | 5.8 ±1.8 | 0.86 | 9.2±1.0 | 0.20 ±0.02 | 3.4±0.4 | 0.99 | 2.7 ±0.2 | 0.13±0.04 | 5.4 ±2.1 | 0.89 |
| **160±20 (1)** | 8.7 ±0.6 | 0.14 ±0.03 | 4.9 ±1.3 | 0.89 | 3.3±1.2 | 0.18 ±0.05 | 11±7.0 | 0.92 | 0.16 ±0.07 | 0.18±0.09 | 3.9 ±2.7 | 0.89 |
| **160±20 (2)** | 9.4 ±0.6 | 0.07 ±0.02 | 9.3 ±2.9 | 0.86 | 2.0±1.2 | 0.08 ±0.03 | 8.2±3.0 | 0.91 | 0.23 ±0.07 | 0.17±0.09 | 4.0 ±2.9 | 0.79 |
| **80±2.5 1)** | 13.7 ±0.3 | 0.12 ±0.03 | 5.8 ±1.8 | 0.86 | 1.0±0.4 | 0.20 ±0.02 | 8.7±5.0 | 0.99 | 5.3 ±3.0 | 0.10±0.04 | 6.8 ±2.8 | 0.89 |
| **80±2.5 (2)** | 13.4 ±0.3 | 0.14 ±0.04 | 5.0±1.8 | 0.83 | 0.6±0.4 | 0.08 ±0.03 | 8.6±6.0 | 0.69 | 8.0 ±3.0 | 0.11±0.06 | 6.1 ±4.4 | 0.78 |

The growth rate (r), doubling time (T_d_), and correlation coefficient (R^2^) values for *Chloromonas brevispina (CB),* *D salina (DS)* and *Chlorella vulgaris (CV)* were calculated via Best-fit exponential models fit to averages of duplicate cell count data of algae cultures spanning 0 to 33-62 days of incubation. The uncertainties on the lag phase duration (LPD) represent the range between the two cell counts of each experiment, whereas the uncertainties on doubling time and growth rate are the standard error of the fit to the average of two cell counts.

**

Figure S27. Comparison of growth curves of *Chloromonas brevispina (CB), Chlorella vulgaris (CV)* and *Dunaliella salina (DS*) at 80 ± 2.5 mbar plotted as a mean value (n=4) of cell count measurements per ml. Error bars are the standard deviation of mean cell counts values.

Figure S28. Comparison of growth curves of *Chloromonas brevispina (CB), Chlorella vulgaris (CV)* and *Dunaliella salina (DS)* at 160 ± 20 mbar plotted as a mean value (n=4) of cell count measurements per ml. Error bars are the standard deviation of mean cell counts values. Larger variations in later time points might be because some cultures are still growing, and some reached death phase.
